# A Unified Comparison of Stimulus-Driven, Endogenous Mandatory and ‘Free Choice’ Saccades

**DOI:** 10.1371/journal.pone.0088990

**Published:** 2014-02-20

**Authors:** Andrei Gorea, Delphine Rider, Qing Yang

**Affiliations:** Laboratoire Psychologie de la Perception, Université Paris Descartes & CNRS, Paris, France; Barrow Neurological Institute, United States of America

## Abstract

It has been claimed that saccades arising from the three saccade triggering modes–stimulus-driven, endogenous mandatory and ‘free choice’–are driven by distinct mechanisms. We tested this claim by instructing observers to saccade from a white or black fixation disc to a same polarity (white or black) disc flashed for 100 or 200 ms presented either alone (Exo), or together with an opposite (Endo) or same (EndoFC) polarity disc (blocked and mixed sessions). Target(s) and distractor were presented at three inter-stimulus intervals (ISIs) relative to the fixation offset (ISI: −200, 0, +200 ms) and were displayed at random locations within a 4°-to-6° eccentricity range. The statistical analysis showed a global saccade triggering mode effect on saccade reaction times (SRTs) with Endo and EndoFC SRTs longer by about 27 ms than Exo-triggered ones but no effect for the Endo-EndoFC comparison. SRTs depended on both ISI (the “gap-effect”), and target duration. Bimodal best fits of the SRT-distributions were found in 65% of cases with their count not different across the three triggering modes. Percentages of saccades in the ‘fast’ and ‘slow’ ranges of bimodal fits did not depend on the triggering modes either. Bimodality tests failed to assert a significant difference between these modes. An analysis of the timing of a putative inhibition by the distractor (Endo) or by the duplicated target (EndoFC) yielded no significant difference between Endo and EndoFC saccades but showed a significant shortening with ISI similar to the SRT shortening suggesting that the distractor-target mutual inhibition is itself inhibited by ‘fixation’ neurons. While other experimental paradigms may well sustain claims of distinct mechanisms subtending the three saccade triggering modes, as here defined reflexive and voluntary saccades appear to differ primarily in the effectiveness with which inhibitory processes slow down the initial fast rise of the saccade triggering signal.

## Introduction

Typically, saccade generation modes are parted in two classes, reflexive (exogenous or stimulus-driven; hereafter referred to as ‘Exo’) and voluntary (endogenously driven) [1]–[6]. The latter class can and perhaps should be parted in two subclasses, namely endogenous mandatory (i.e. instructions based; ‘Endo’) and ‘freely-chosen’ (‘EndoFC’ [7]). In turn, Endo saccade studies can also be partitioned in three major classes. In one such class saccades are directed in accordance with a foveally presented symbolic cue (such as an arrow [6], [8], or with a pre-specified target attribute (such as shape or color; e.g., [9], [11] or target location [11], [12]. Other Endo saccade studies focus on saccades to the memorized location of an extinguished target (memory guided; e.g., [13]. Anti-saccades – i.e., to a location opposite and symmetrical to that of a transient target – form a third Endo saccade class [5], [14]. EndoFCs saccades can be roughly parted in visually guided (as in visual search or free scene exploration [15]) and spontaneously triggered in the dark [16].

The characteristics of saccades within these classes and their subclasses may differ quite drastically (particularly in latency [4], [6], [11], [12], [17], [18]), can be modified selectively by localized cortical lesions in humans [19]–[21] as in monkeys [22], and appear to be generated in distinct cortical areas (the intraparietal sulcus, the precuneus, the angular gyrus and the posterior cingulated cortex dominantly involved in the generation of reflexive saccades and the frontal eye fields, the dorsolateral prefrontal cortex the supplementary eye fields, the pre-supplementary motor area and the anterior cingulated cortex mostly involved with voluntary saccades; see reviews in [4], [6], [7], [23]–[25]. With a few exceptions (including studies having compared, in addition to reflexive saccades, memory-guided and anti-saccades [26], or memory-guided and multiple saccades along an instructed path [15], such comparisons involved only two saccade generation modes and focused on their mean/median latencies (saccade response time, SRT). Symbolically directed saccades (typically classified as Endo) were found to be ∼100 ms [4] and ∼60 ms [6] slower than Exo saccades. Nachev et al. [18] found that EndoFC saccades are triggered about 40 ms later than symbolically directed ones. In all these studies the two saccades types were randomly interleaved. None of them presented or even alluded to the SRT-distributions. Typical exceptions are studies mostly focused on the gap-effect (yielding ‘express’ saccades with SRTs ≤ ∼100 ms [27], [28]) and on distractor-induced saccade inhibition [12], [14], [29], [30]. Save for such exceptions, and despite prior reports of N-modal SRT-distributions (with N up to 4) in single target saccade tasks under both gap and overlap conditions [31], the saccadic behavior under the three triggering modes was essentially characterized in terms of its mean/median latency. When occasionally reported for Endo-type, non-gap conditions [32], bimodal SRT-distributions show peaks around 150 ms (significantly beyond the standard express saccades range [33]) and 300 ms but such bimodality was apparent for only 5 out of 15 observers. On visual inspection, SRT-distributions reported by Bompas & Sumner [29] for target alone and target+distractor conditions (Exo- and Endo-type, respectively; see their Fig. 3) also show clear inter-observers differences and fail to display an obvious uni- vs. bimodal shape. No such bimodality appears in the SRT distributions (cumulated over observers) reported by Walker et al. [11], [12] for target-distractor tasks.

Leach & Carpenter [17] presented SRT-distributions (reciprobit latency plots) for saccades triggered by 100 ms targets presented either as singletons (Exo condition), or by pairs with stimulus onset asynchronies (SOAs) between 20 and 80 ms. In the latter case observers were instructed “to look, as quickly as possible, at whichever target caught their attention” (p. 3439), a speeded temporal order judgment (TOJ). As TOJ performances are practically at chance level for 20 ms SOAs [34], this condition could be assimilated to an EndoFC type (with the restriction that observers were instructed to saccade “as quickly as possible”, an instruction that might counteract the putative effects of a ‘free choice’ decision). Contrary to predictions of a simple race model [34], [35], such pseudo-EndoFC saccades were overall 30 ms slower than for the Exo condition, an outcome accounted for by mutual inhibition between decision units [14], [17], [36]–[38]. Except for such a global latency shift (repeatedly reported in the presence of a distractor; see below), Leach & Carpenter’s [17] Exo and pseudo-EndoFC SRT-distributions were similar and unimodal. (Unfortunately, the SRTs considered for the asynchronous targets were cumulated over all SOAs potentially blurring a possible bimodality as well as preventing the isolation of the condition closest to a ‘true’ EndoFC one).

While the three saccade triggering modes (Exo, Endo and EndoFC) may well address different neural substrates, their efferent signals ultimately target the intermediate layers of the superior colliculus (SC) where they are assumed to be dynamically integrated through lateral excitatory-inhibitory interaction fields (see reviews in [38]–[40]). Inasmuch as ‘purely’ reflexive (Exo) saccades are thought of as being made in the total absence of a volitional/intentional signal (and assuming no gap-effect), the unimodality of their latency distributions is to be expected. Instead, Endo and EndoFC triggering modes may not be immune to the intrusion of reflexively triggered saccades, and if so, their SRT-distributions should present a more or less pronounced bimodal aspect. As bi- (or tri-)mode SRT-distributions were occasionally reported even with standard reflexive tasks [27], [29], [31], [41], [42], it is tempting to conclude that a strict reflexive-voluntary separation may not be possible. In all events, it can be conjectured that the reflexive-voluntary mixture should be enhanced if the three triggering modes were randomly interleaved. We therefore assessed the saccadic behavior under both blocked and mixed condition.

As Endo and EndoFC modes involve the presence of an accompanying stimulation (distractor or alternative target), the respective SRTs are presumably affected by an inhibitory process from units activated by the distractor (Endo) or by the accompanying target (EndoFC [11], [12], [36]). Whether the timing characteristics of such inhibition and its efficacy are the same in Endo and EndoFC modes is a question the present study answers positively. Saccade triggering units are also inhibited by collicular ‘fixation’ neurons inasmuch as the delay between the fixation offset and the target(s) onset is shorter than 200–300 ms (the gap effect [28], [38], [43]–[45]). We therefore also manipulated this delay to see whether the two inhibitory processes (in-between saccade triggering units and in-between fixation and saccade triggering units) interact thereby differentially impacting the saccadic behavior under Endo and EndoFC conditions.

**Figure 1:**
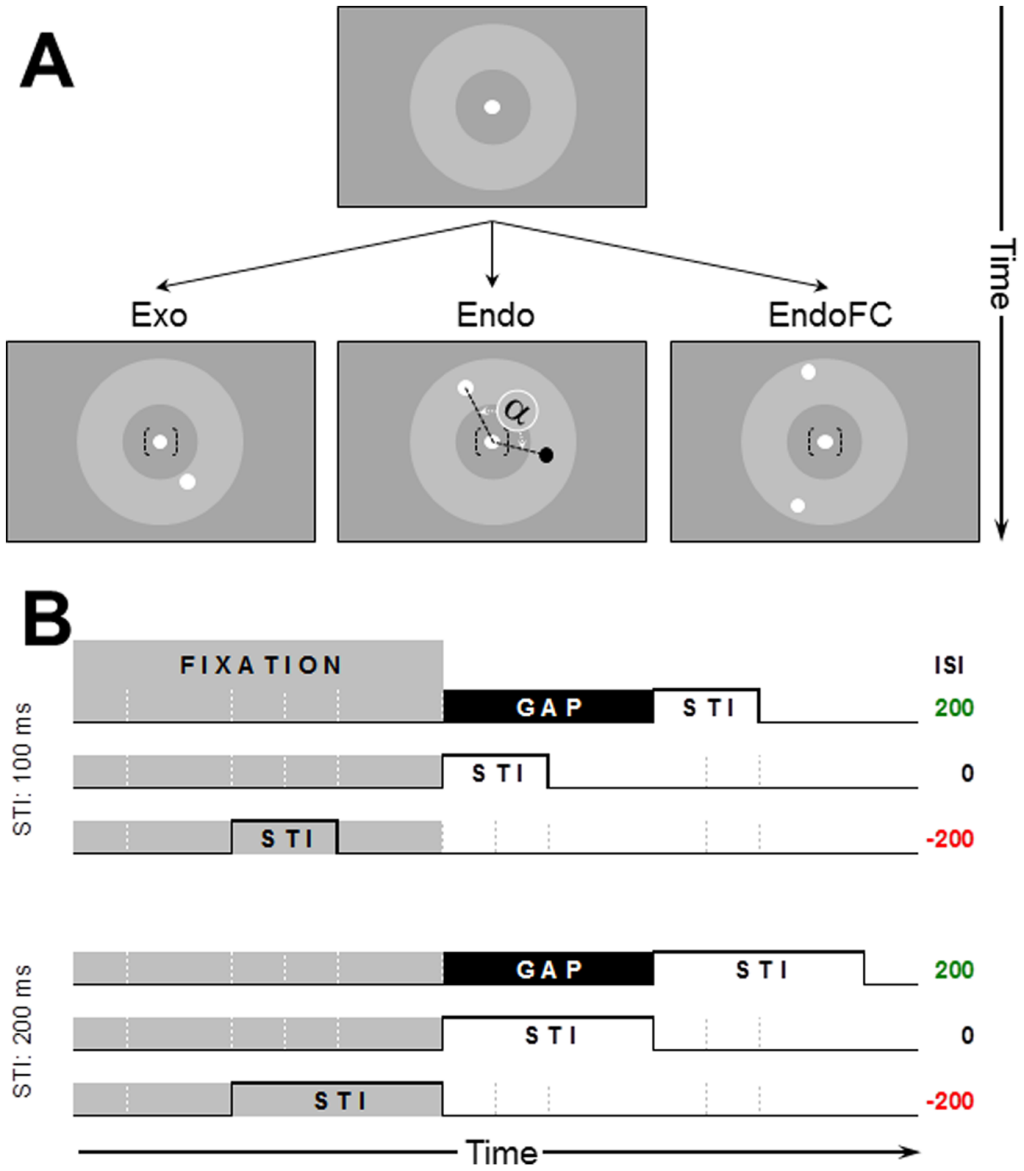
Spatial and temporal layout of the different experimental conditions. **A.** Spatial layout showing fixation (top panel) and target and distractor discs. Fixation in the bottom panel is shown between brackets because, depending on the timing condition, it was or was not present during the display of the target(s)/distractor. The target disc was always of the same color as the fixation disc and was presented by itself (Exo), accompanied by an opposite polarity (Endo) or by a same polarity (EndoFC) disc. Target(s) and distractor where presented at the same eccentricity randomized across trials within the 4-to-6° area indicated by the gray annulus (invisible in the experiments). Target and distractor (Endo) or the two potential targets (EndoFC) were separated by a minimum angle α of 90°. **B.** Time-sequence of one trial with 100 and 200 ms stimuli (top and bottom panels) for each of the three fixation-target(s)/distractor intervals: gap, simultaneous and overlap conditions (ISI = 200, 0, −200, respectively).

The present study compares for the first time Exo, Endo and EndoFC saccades (as defined below) with a unified experimental paradigm. It focuses on their mean latencies and their latency distributions. Observers had to saccade to a shortly flashed white (or black) disc/target when presented (a) alone (Exo), (b) together with a black (or white) disc/distractor (Endo) or (c) with another white (or black) disc/2^nd^ target (EndoFC) (see Figure 1A). Target’s color was announced by the color of the fixation disc and observers were instructed to freely choose between the same color targets (EndoFC). The three conditions were tested both under blocked and mixed designs for target durations of 100 and 200 ms and for conditions where the fixation and target/distractor discs (a) overlapped in time for 100 and 200 ms or were separated by a (b) 0 or (c) 200 ms gaps.

The present empirical characterization of the three saccade triggering modes can be and has been challenged (by reviewers) on grounds that they do not capture a valid graduation between reflexive and voluntary saccades. As already noted, the present definition of the Endo mode is strictly equivalent to that of a target+distractor trial used in many studies having assessed the distractor effect in reference to a ‘no distractor’ condition, itself equivalent to the present definition of the Exo mode. Our rationale for referring to the target+distractor stimulus-configuration as an Endo triggering mode stems from the notion that when two different stimuli are presented, observers need to make a discrimination decision prior to directing their gaze to the stimulus with a pre-specified characteristic (the target). Such discriminatory decision based on stored specifications must be taken in non-visual areas so that the ensuing saccades qualify as endogenously triggered. Pre-specifying the feature(s) of a saccade target was frequently used to discriminate them from “reflexive” (or “exo”) ones [9]–[11]. The rationale underlying our empirical definition of the EndoFC triggering mode is rather obvious: once having assessed the equivalence of the two targets, the observer is free to choose either of them, a process involving a “second order” decision. Precisely because we are aware that the theoretical and empirical distinction between voluntary and involuntary actions raises insoluble philosophical problems [18], [24] and because different studies used different, and potentially arbitrary stimulation conditions to isolate them, we believe that the presently proposed empirical definitions are no less valid than prior ones, while presenting the notable advantage of allowing the unitary study of the three saccade triggering modes with a coherent family of stimulation conditions. We acknowledge however that different experimental paradigms may lead to results and conclusions different from the present ones.

## Methods

This study was conducted in agreement with the requirements of the Helsinki convention and approved by the local ethical committee of Université Paris Descartes. Participants provided their written informed consent to participate in this study. Their consent was approved by this committee.

### Stimuli

Participants were seated in a dimly lit room with the head positioned on a chin rest ∼60 cm from a 38°×28° 22W Formac ProNitron 22800 screen with a spatial resolution of 1440 by 1050 pixels and a 100 Hz refresh rate. Movements of the right eye were measured using an EyeLink 1000 Desktop Mount (SR Research, Osgoode, Ontario, Canada) with an average spatial resolution of 0.25°, and a 1 kHz sampling rate. The experiment was controlled by an Apple MacProDual Intel-Core Xeon computer. The experimental software controlling the stimuli display and response recordings was implemented in MATLAB using the Psychophysics toolbox [46], [47] and EyeLink toolbox [48]. Fixation was specified by a white (29 cd/m^2^) or black (0.04 cd/m^2^) 0.3° disc. The stimuli were also 0.3° black and/or white discs presented either one or two at a time within an annulus centered on fixation with 4° and 6° radii (see Figure 1A). When two discs were presented they were displayed at the same eccentricity and always separated by more than 90 degrees on a virtual circle so as to have them projected on either left-right or up-down hemifields. The color of the fixation disc announced the color of the saccade target so that for trials displaying one single disc or two same color discs their color necessarily matched the color of the fixation disc.

### Procedure

Trials yielding (i) saccade latencies longer than 500 ms (0.7%), (ii) landing positions more than 2.5° away from the target (almost exclusively saccades directed to the distractor, in the Endo condition; 3.8 and 15.8% in the Blocked and Mixed conditions, respectively) were detected online, discarded and repeated at the end of each block. Additional rejections were performed off-line on the Eyelink edf files if (i) a blink was detected within 700 ms from the onset of the target and if (ii) the first saccade amplitude was less than 60% of the distance between fixation and target thus excluding cases where the target was reached via corrective saccades.

Each trial was initiated by the observer’s key-press. Once their eye was detected within a 1.5° window around fixation for 300 ms, one (Exo) or two discs (different colors: Endo; same color: EndoFC; saccade triggering type, ST factor) were displayed after a random (1000–1500 ms) interval for 100 or 200 ms (Target Duration, TD factor). Their onset relative to the fixation offset (ISI factor) was 200 ms before (overlap condition), simultaneous or 200 ms after (gap condition; see Fig. 1B). Each of the 6 temporal display conditions were run in separate blocks, while the ST conditions were run in both blocked and mixed designs (D factor), so that altogether each observer run 2[TD]×3[ISI]×3[ST]×2 [Blocked/Mixed] = 36 distinct blocks of 50 trials each repeated once, i.e. a total of 3,600 trials per observer.

Before the start of each block of trials observers were informed about the block type they were about to run. They were instructed to make speeded saccades to the disc of the same color as the fixation disc and, when presented with two discs of the same color, to choose freely their saccade target. The color of the fixation/target disc(s) was randomized across trials as well as the location of the target and distractor stimuli (with the constraints specified above). The whole experiment was completed in about 3 hours per observer and run over 2–3 days.

### Observers

Five observers (three male and two female, between 26 and 60 years old) including the three authors completed the whole experiment. They had ample experience with eye-movement recordings. They were given at least 50 trials training with each of the specific conditions to be used thereafter.

## Results

### Errors

When excluding saccades directed to the distractors (only present in the Endo mode; 3.8 and 15.8% in the blocked and mixed conditions respectively) rejections based on landing errors (more than 2.5° away from the target and post-hoc rejections of saccades with amplitudes less than 60% of the distance between fixation and target; see Procedure) were less than 2% in all the remaining conditions.

### Saccade Latencies

**Figure 2:**
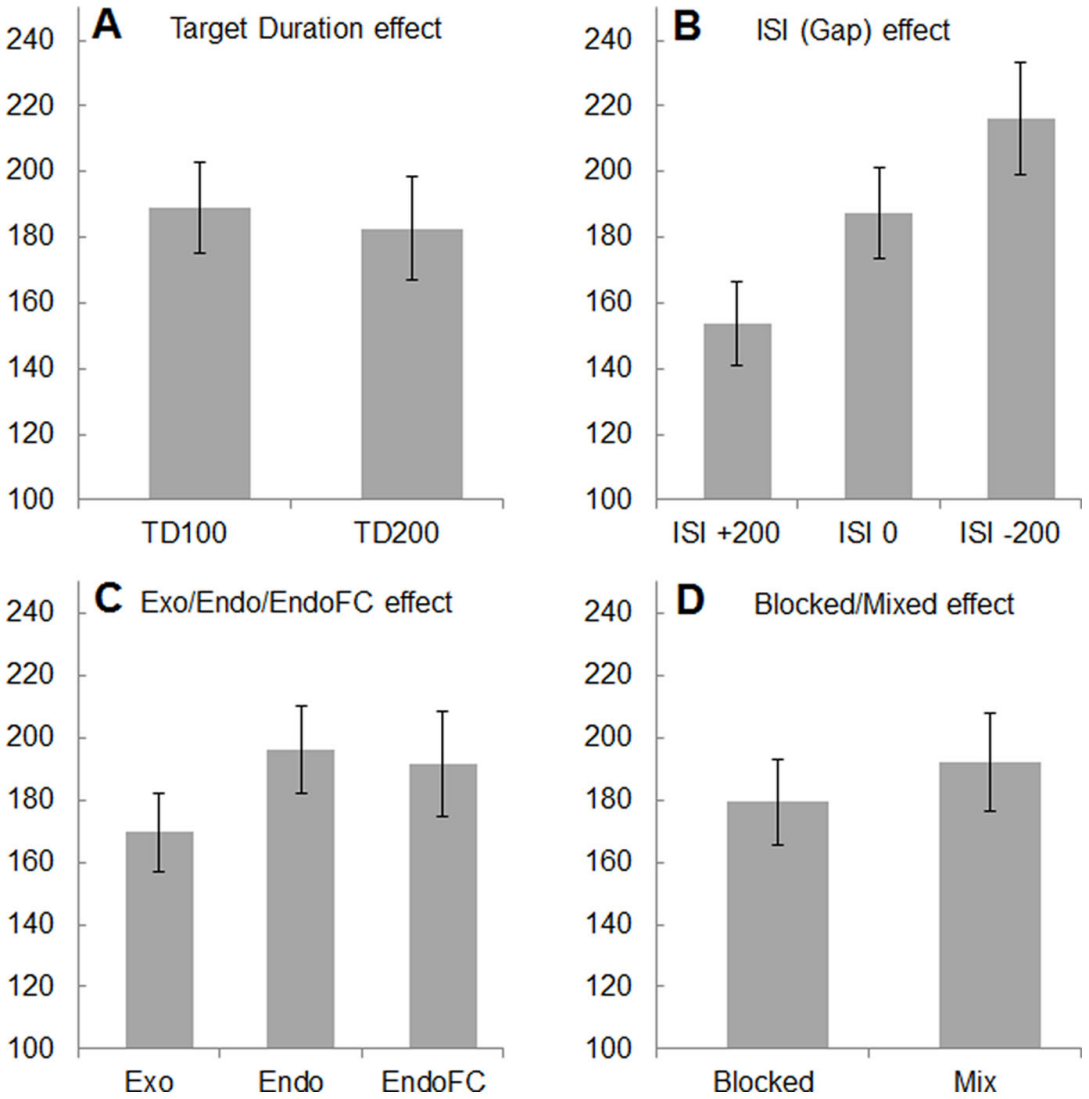
SRTs averaged over observers and conditions to show the effects of Target Duration (A), ISI (B), Saccade Triggering modes (C) and experimental Design (D). Thin bars are ±1 SE over the 5 observers.

Figure 2 illustrates the effects of the four main factors presently studied, namely TD (2 modalities: 100, 200 ms), ISI (3 modalities: −200, 0, 200 ms), ST (3 modalities: Exo, Endo, EndoFC) and D (2 modalities: Blocked, Mixed) (see Procedure). Two 4-way ANOVA with repeated measures run on the SRT means and medians confirm what can be inferred from mere inspection (the two numbers given correspond to mean/median analyses; Geisser-Greenhouse correction for sphericity are signaled by an asterisk): with the exception of the D factor whose effect (13 ms) fringes significance (F(1,4) = 6.20/5.43, p = 0.067/0.080), the remaining three factors yield highly significant effects (TD: F(1,4) = 60.51/6.87, p = 0.0015/0.059; ISI: F(2,8) = 16.64/9.28, p = 0.014*/0.037*; ST: F(2,8) = 70.76/30.78, p = 0.000118*/0.0023). With one exception (ST×TD: F(2,8) = 6.30/6.02, p = 0.036*/0.069), none of the two- to four-way interactions was significant.

**Figure 3:**
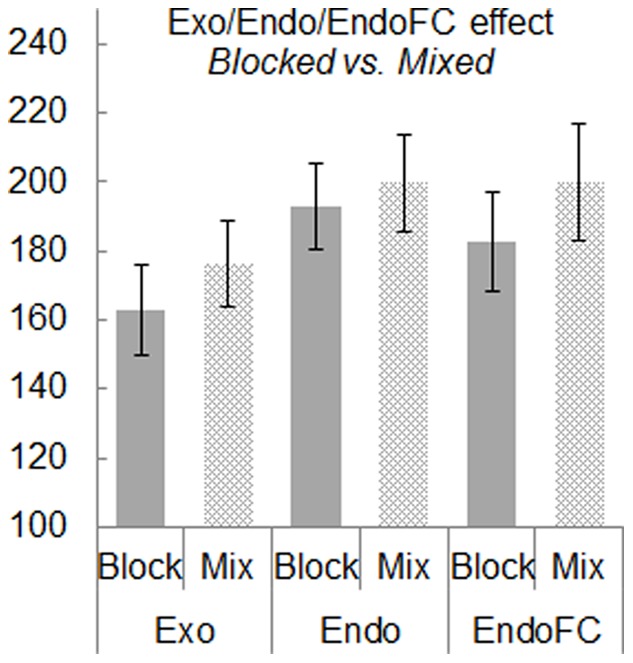
Mean Exo, Endo and EndoFC SRTs under blocked and mixed conditions.

The TD effect (10 ms) is in principle accountable in terms of the target brightness as, due to temporal integration, the 200 ms target is more ‘energetic’ than the 100 ms one [49]. The ISI effect is well known in the literature as the *gap-effect*. Its present magnitude for a 200 ms gap compared to a 0 ms gap (30 ms) and to a 200 ms overlap (64 ms) is in the range of previous reports [27], [33], [43]. Of main interest in the present study is the ST effect. While a post-hoc Tukey test shows no significant difference between Endo and EndoFC conditions, the difference between Exo, on the one hand, and Endo+EndoFC on the other is highly significant (F(1,4) = 362.64/19.04, p = .000045/0.012). The Endo-EndoFC difference becomes (close to) significant, however, when tested for the blocked sessions only (with EndoFC SRTs *shorter* than Endo SRTs – see Figure 3; Tukey; p = 0.066/0.022), most likely because in the blocked EndoFC design observers could associate a future target with a pre-selected hemifield (left/right or up/down) thereby occasionally skirting an online ‘free choice’. Such strategy was confirmed for at least some proportion of trials by two observers’ casual introspective reports. It should be also noted that the 3.8% and 15.8% (blocked and mixed conditions, respectively) misdirected Endo saccades (signaled by a tone, rejected and repeated at the end of each session) might have encouraged observers to lengthen their SRTs. While there is no way to test this conjecture, for Endo SRTs to be significantly shorter than EndoFC SRTs, such putative artificial lengthening of the former must have been of at least 35 ms in average, an unlikely event. Finally, the ST×TD interaction (not significant when tested on the median SRTs) might well be the consequence of the fact that decreasing stimulus duration from 200 to 100 ms is less critical for the fastest (Exo) than for the slower (Endo, EndoFC) saccades as the latter require longer stimulus processing time for reaching a decision.

### Latency Distributions Analysis

The analysis of the SRT distributions followed two distinct approaches. The first approach was grounded on the notion that ‘reflexive’ (Exo) and ‘voluntary’ (Endo & EndoFC) saccades are triggered at distinct neural loci (see the Introduction). The tentative conjecture was that the reflexive/voluntary distinction might be less clear-cut than previously suggested as the respective SRT-distributions might each consist of a mixture of ‘pure’ reflexive and ‘pure’ voluntary saccades, perhaps in different proportions. If so, SRT-distributions should be in general bimodal with the weights/proportions of the two saccade types modulated by their triggering modes, i.e. with stronger weights for (larger proportion of) reflexive (fast) and voluntary (slower) saccades under the present Exo and Endo or EndoFC conditions, respectively. (As noted in the Introduction, three-modal (or more) SRT-distributions were also mentioned and theorized in the literature [50] but their low reproducibility (across observers and experimental conditions) together with their debatable “three-loop” theoretical account [51] made us drop an analysis of the data along these lines).

The conjecture was tested by fitting uni- and bimodal log-normal Gaussians (in fact the log-SRTs were fit with normal functions) to each observer’s SRT distribution under each of the 36 experimental conditions (available on request) as well as to the vincentized data [52], [53] for each of these conditions. The fits were obtained with the Matlab *gmdistribution.fit(X,k)* function (an Expectation Maximization algorithm used to construct an object *obj* of the gmdistribution class containing maximum likelihood estimates of the parameters in a Gaussian mixture model with k components). The goodness of fit of the unimodal vs. bimodal models was assessed with the Akaike information criterion (AIC [54]. The proportion of ‘fast’ (and ‘slow’) saccades for the three triggering modes was also assessed. A second test of the SRT-distributions bimodality consisted in measuring Sarle’s bimodality coefficient, *β* [55], [56] combined with the *excess kurtosis* test [57] (see below).

The second approach to addressing the putative distinction between SRT-distributions under the present Exo, Endo and EndoFC conditions capitalized on the inhibition of saccades by a distractor (Endo) or by the alternative target (EndoFC) [11], [12], [14], [29], [30]. Such inhibition is supposed to produce a dip in the SRT-distributions within the range of delays requested for inhibition to be efficient. In this analysis we looked for a putative difference in the timing of the inhibitory process under Endo and EndoFC conditions. To estimate the amplitude and timing of such dips we applied the same method as [29]. It consisted in calculating, for each 10 ms time-bin (the use of smaller bins would have entailed too many bins with no saccades) the proportional change of the saccades number in the Endo and EndoFC SRT-distributions relative to their number in the corresponding baseline Exo SRT-distribution, i.e., (baseline – distractor)/baseline, the *distraction ratio*. The time-bin with the largest distraction ratio (largest dip amplitude) is taken to be the time-bin where inhibition is most effective. The beginning of the dip was obtained by going backward in time from that time-point until the ratio became smaller than 5% (or the no-distractor bin was empty). To improve stability of these estimates, distributions were smoothed using a Gaussian kernel with 7 ms window and 1 ms SD and interpolated to obtain 1 ms precision, before the ratio was calculated.

Given the present empirical characterization of the three saccade triggering modes, the two conjectures above (mixture of reflexive and voluntary saccades vs. inhibition by distractor, both applicable to the Endo and EndoFC triggering modes) cannot be disentangled. It is not preposterous to posit that the critical difference between reflexive and voluntary saccades consists in that only the latter are reliant on an inhibitory process which is either absent or too slow to affect reflexive saccades [29].

### Uni- and Bimodal Gaussian Fits

**Figure 4:**
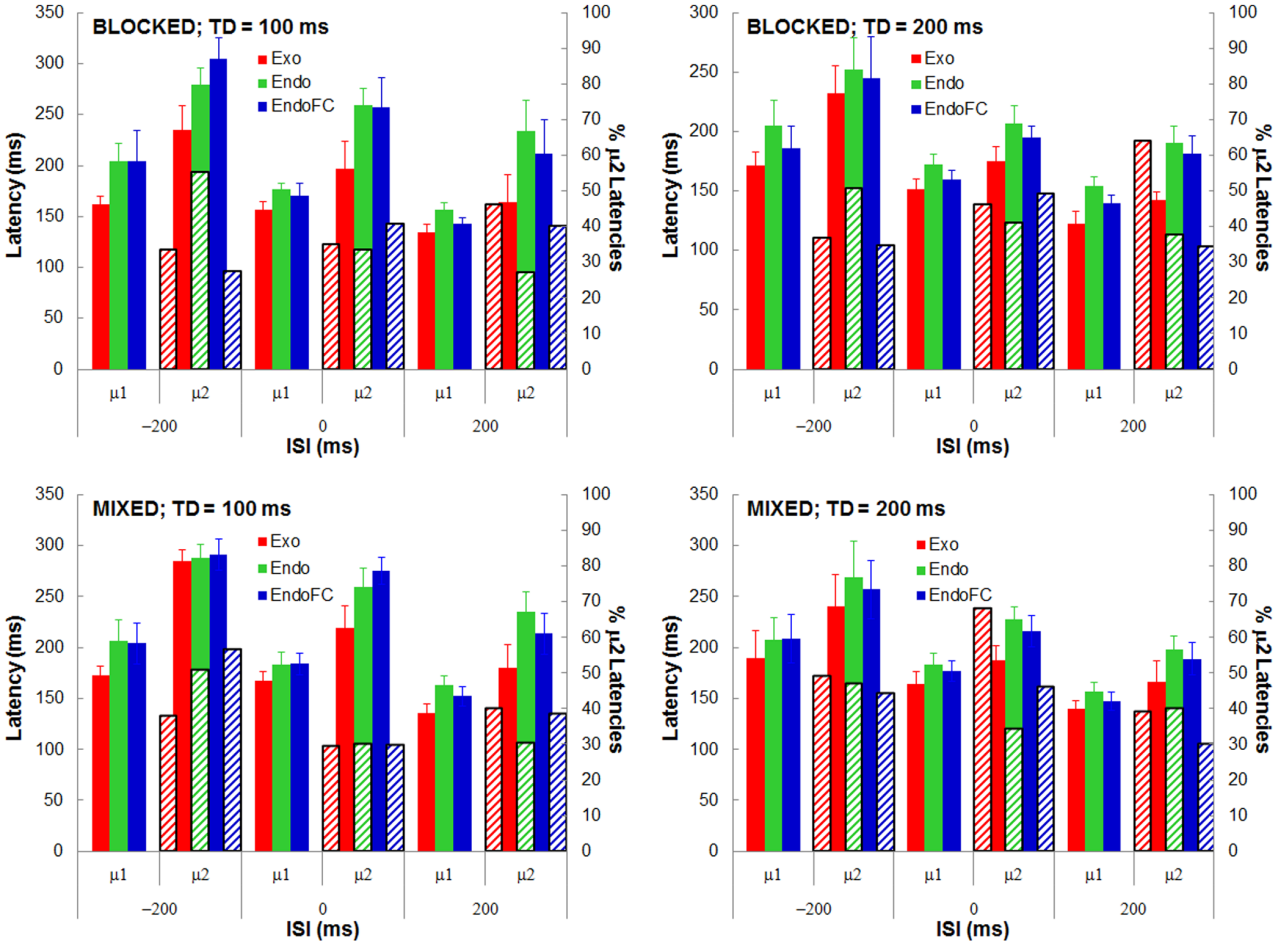
Means across observers (μ1, μ2) of the bimodal SRT-distribution fits (left ordinates, solid bars) and percentages of saccades in the slower SRT-distributions (right ordinates and striped bars) for the blocked and mixed designs (upper and lower panels), the two target durations (left and right panels) and for each of the three triggering modes (Exo: red; Endo, green; EndoFC, blue) as a function of ISI.

1. Over the 2 designs (blocked and mixed), the 6 timing conditions and the 5 observers (i.e. out of 60 cases per saccade triggering mode), the AIC favored the bimodal fits in 34, 42 and 41 cases for the Exo, Endo and EndoFC conditions, respectively. None of the three χ^2^ values for comparisons by pairs reached significance.

(2) Figure 4 shows the mean latencies (μ; solid bars, left ordinate) of the bimodal fits (including the cases where the AIC favored the unimodal fit; their exclusion would have unbalanced the number of observers per condition leading to non-representative means and rendering an ANOVA impossible) together with the estimated proportions of the longer latencies (right ordinate; textured bars) averaged over the five observers for the blocked and mixed designs (top and bottom panels), for each of the 6 timing conditions (abscissae) and for the three saccade triggering modes (Exo: red; Endo: green; EndoFC: blue). A 5-way ANOVA including (in addition to the 4 factors specified in the *Saccade latency* section) the “Mean” factor (two modalities: μ1 and μ2), shows, as expected, the same significant effects as the 4-way ANOVA on the global means (p = .04,.001,.00009, for factors TD, ISI and ST, respectively) with no significant difference between fast Endo and fast EndoFC fast (μ1) or between slow Endo and slow EndoFC (μ2) saccades. In addition, a 4-way ANOVA with repeated measures (TD[2]×ISI[3]×ST[3]×D[2]) performed on the normalized percentages X (in fact, on 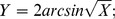 [58], p. 408), of the slow saccades shows no significant effect of any of the four factors. In short, the characteristics of the bimodal fits do not appear to discriminate between the three saccade triggering modes other than confirming the global mean analysis having showed that Endo and EndoFC saccades are significantly longer than Exo saccades.

**Figure 5:**
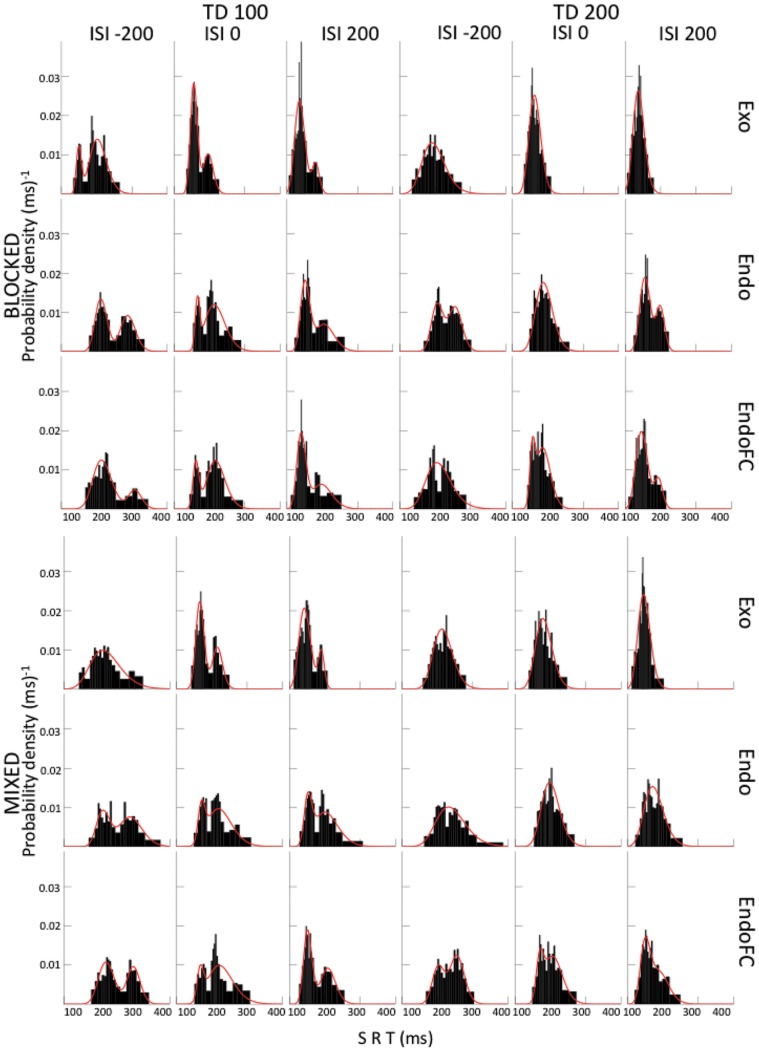
Vincentized SRTs (5 observers; bars) and their uni- or bimodal log-normal fits (continuous curves) for all experimental conditions. As attested by their summary statistics given in Table 1, the uni- vs. bimodal fits show no obvious structured pattern.

Figure 5 displays the vincentized SRT-distributions of the 5 observers aggregated together for each of the 36 experimental conditions together with their best log-normal uni- or bimodal fits. Table 1 presents the means of the bimodal log-normal fits for each of these conditions. Fig. 5 is intended to show the large variability and unsystematic aspect of these distributions across experimental conditions. The same variability was observed across observers for each of the 36 experimental conditions so that it is meaningless to present a “representative observer”. The SRT-distributions for each of the 5 observers and for all experimental conditions are presented in the Supplementary Information section (Figures S1-6B and S1-6M).

**Table 1:** Means (μ) and standard errors (SE) of the bimodal log-normal fits of the vincentized SRT-distributions together with the proportion of the faster (μ1) saccades shown separately for Blocked and Mixed designs as well as averaged over these two designs.

		Exo						Endo						EndoFC					
		Blocked			Mixed			Blocked			Mixed			Blocked			Mixed		
		μ1	μ2	p1	μ1	μ2	p1	μ1	μ2	p1	μ1	μ2	p1	μ1	μ2	p1	μ1	μ2	p1
	ISI −200	149	202	0.25	211	––	1.00	212	287	0.62	211	288	0.50	213	308	0.84	219	297	0.66
TD 100	ISI 0	155	196	0.72	166	215	0.69	166	213	0.23	171	219	0.26	161	216	0.30	167	221	0.22
	ISI +200	135	182	0.86	142	190	0.86	151	205	0.64	152	201	0.37	140	198	0.67	152	209	0.62
	ISI −200	191	––	1.00	212	––	1.00	207	258	0.57	231	––	1.00	205	––	1.00	203	256	0.49
TD 200	ISI 0	162	––	1.00	179	––	1.00	187	––	1.00	198	––	1.00	156	186	0.30	170	207	0.30
	ISI +200	136	––	1.00	145	––	1.00	155	199	0.75	170	––	1.00	145	194	0.85	149	191	0.60
MEANS		**155**	**193**	**0.81**	**176**	**203**	**0.93**	**180**	**232**	**0.64**	**189**	**236**	**0.69**	**170**	**220**	**0.66**	**177**	**230**	**0.48**
*SE*		*8*	*4*	*0.12*	*13*	*7*	*0.05*	*11*	*16*	*0.10*	*12*	*19*	*0.14*	*13*	*20*	*0.12*	*12*	*16*	*0.07*
Blocked & Mixed		**μ1**			**μ2**		**p1**	**μ1**			**μ2**		**p1**	**μ1**			**μ2**		**p1**
GLOBAL MEANS		**165**			**197**		**0.87**	**184**			**234**		**0.66**	**173**			**226**		**0.57**
*SE*		*8*			*28*		*0.06*	*8*			*36*		*0.08*	*8*			*34*		*0.07*
RAW GLOBAL MEANS		**181**						**209**						**200**					
WEIGHTED GLOB. MEANS		**172**						**203**						**195**					

The vincentized data confirm the general trends unveiled by the two statistical analyses performed on the global SRT means and on the SRT means obtained from the bimodal SRTs fits. Of particular interest are the means (weighted by their proportions) for the Exo (172 ms), Endo (203 ms) and EndoFC (195 ms) saccade triggering modes showing a presumably significant difference between Exo and the remaining two modes with no significant difference Endo and EndoFC.

### Sarle’s Bimodality Coefficient (β) and Kurtosis Test

The bimodality coefficient *β* lies between 0 and 1 (see above). Values greater than 5/9 (.555) are suggestive of a bimodal (or multimodal distribution). Over the blocked and mixed designs, the 6 timing conditions and the 5 observers (i.e. out of 60 cases per saccade triggering mode), the “*β* >.555” count was 15, 16 and 14 for Exo, Endo and EndoFC conditions, respectively, clearly not statistically different. To be noted, the total *β* >.555 count (45) is much smaller than the bimodality count (117) as attested by the AIC when comparing uni- and bimodal fits of the SRT-distributions. The *β* >.555 count for Blocked and Mixed conditions was 23 and 22, respectively, hence not supporting the conjecture that the mixed design would promote bimodality.

A necessary but not sufficient condition for a symmetrical distribution (in our case lognormal) to be bimodal is that its kurtosis (the standardized fourth moment around the mean) be less than 3 (see Appendix in [57]). Out of a total of 60 counts per saccade triggering mode, there were only 13, 18 and 13 such cases for Exo, Endo and EndoFC conditions, respectively, once again not statistically different. Moreover, there were altogether only 9 cases were *β* and kurtosis were simultaneously indicative of bimodality. Again, the excessive kurtosis count did not differ significantly between Blocked and Mixed designs (72 and 65, respectively).

### Distractor Inhibition Characteristics

Using Bompas & Sumner’s [29] technique described above, we identified the timing of the start and of the apex of inhibition presumably induced by the non-target disc (i.e. distractor in Endo and alternative target in EndoFC). As it should be expected given that target and non-target discs were presented simultaneously, these inhibition indices typically occur very early in time so that their computation is based on very few saccades and is therefore statistically unreliable (see also Fig. 3 in [29]). Table 2 displays the two time-points averaged over the 5 observers for each of the six timing conditions.

**Table 2:** Derived timings and standard errors of the Start and Maximum inhibition by the distractor or by the twin not-chosen target in the Endo and EndoFC conditions, respectively (as derived from the distraction ratio computation used by Bompas & Sumner, 2011).

		Endo				EndoFC				ALL				ALL by ISI				
		Blocked		Mixed		Blocked		Mixed		Start		Max		Start		Max		
		Start	Max	Start	Max	Start	Max	Start	Max	Mean	*SE*	Mean	*SE*	Mean	*SE*	Mean	*SE*	
	ISI −200	132	133	165	171	146	170	132	133	144	*8*	152	*11*	**139**	*8*	**148**	*10*	ISI −200
TD 100	ISI 0	112	113	109	117	102	115	106	110	107	*2*	114	*1*					
	ISI +200	76	81	82	91	74	97	79	87	78	*2*	89	*3*	**106**	*2*	**111**	*2*	ISI 0
	ISI −200	122	126	147	152	100	108	172	194	135	*16*	145	*19*					
TD 200	ISI 0	104	108	110	111	96	101	108	113	105	*3*	108	*3*	**77**	*2*	**89**	*2*	ISI +200
	ISI +200	82	88	76	97	68	85	78	90	76	*3*	90	*3*					
MEANS		**105**	**108**	**115**	**123**	**98**	**113**	**113**	**121**	**107**		**116**						
*SE*		*9*	*8*	*14*	*13*	*11*	*12*	*14*	*16*	*11*		*11*						
		Endo				EndoFC												
Blocked & Mixed		Start		Max		Start		Max										
GLOBAL MEANS		**110**		**116**		**105**		**117**										
*SE*		*8*		*8*		*9*		*10*										

The implication of the obvious equivalence of the two time-points of the inhibition indices for the Endo (110, 116) and EndoFC (105, 117) conditions is that the assumed inhibitory processes contributing to saccades initiation under these two triggering modes are undistinguishable. A 5-way ANOVA (including the ‘Start-Max’ factor) confirms this observation. It also shows a significant effect of the ISI factor (F(2,8) = 4.97, p = 0.039) pointing to the fact that inhibition kicks in progressively earlier as fixation and target(s) overlap in time (ISI = −200; Start-Max = 139–148 ms), are contiguous (ISI = 0; 106/111 ms) and are separated by a gap (ISI = +200, 77–89 ms). Additional support to the equivalence between Endo and EndoFC saccades comes from the highly significant correlations (r) between the inhibition (start and max) points for the Endo and EndoFC conditions for each observer (Table 3).

**Table 3:** Observer-wise correlations (r) between the inhibition time-points in Endo and EndoFC and their significance levels (*p*).

Observers		AG	DR	JM	VH	QY
**Start**	**r**	**0.63**	**0.70**	**0.84**	**0.92**	**0.88**
	*p*	*0.0010*	*0.0001*	*<0.0001*	*<0.0001*	*<0.0001*
**Max**	**r**	**0.44**	**0.69**	**0.77**	**0.50**	**0.83**
	*p*	*0.0300*	*0.0002*	*<0.0001*	*0.0100*	*<0.0001*

Upper two and lower two rows are for starting (Start) and maximum (Max) inhibition points, respectively.

## Discussion

Using a coherently progressive transition from exogenously triggered (Exo) to mandatory (Endo) and to ‘free choice’ (EndoFC) endogenously triggered saccades under six timing conditions and two experimental designs (blocked and mixed sessions), the present study revealed no significant differences between the three saccade triggering modes (with the exception of shorter SRTs for the Exo than for the Endo and EndoFC modes; see below). It should be pointed out from the start that while such absence of a difference suggests, contrary to prior reports, a common neural origin of the three saccade types by no means does it prove it. The following discussion should be read with this precaution in mind.

The factors presently manipulated that yielded significant SRT effects were the target duration, the time interval between the offset of fixation and onset of the saccade target (ISI; the gap effect) and the saccade triggering mode. The effect of the experimental design (blocked vs. mixed sessions) fringed significance. Inasmuch as the two target durations used (100 and 200 ms) are partly within the temporal integration regime of the visual system (Gorea & Tyler, 1986), the shorter saccades found for the longer target duration (183 vs. 189 ms) are accountable in terms of the higher energy of such targets. The fixation-target delay effect (SRTs 216, 187, 154 for ISIs of −200, 0, +200 ms) essentially replicates the gap-effect [28] whose account in terms of the earlier vs. later disengagement of collicular ‘fixation’ neurons that compete with saccade triggering neurons [38], [39], [43]–[45] is strengthened by the presently observed SRT lengthening under fixation-target temporal overlap conditions. The close to significant SRT lengthening under mixed (192 ms) relative to blocked (179 ms) experimental conditions suggests the existence of a tonic preparatory activity of saccade-related neurons throughout a blocked session presumably of the same type (i.e. low-frequency spiking) as the one observed during the period separating fixation offset from target onset in the standard gap-effect studies [44]. Interestingly, the present data show that such global advanced motor preparatory activity does not necessarily lead to the initiation of *express saccades* as the presently measured SRT-distributions under the gap (ISI = 200 ms) conditions do not display a systematic bimodality with the shortest SRTs of 123±10 ms (Exo conditions, target duration 200 ms), significantly longer than typically reported (∼100 ms; [33]. This is consistent with Dorris et al.’s [44] note that “the increase in the activity of buildup neurons coding for the upcoming target location is necessary for express saccade generation” but that “fixation neuron attenuation is, by itself, insufficient to account for [it]” (p. 8577; see also [59]).

The present study was mainly focused on the dependence of the saccadic behavior on the saccade triggering mode, namely Exo, Endo and EndoFC. Two aspects of this behavior were studied: its mean/median latency (SRT) and the statistical properties of these latencies, i.e. their distributions. To our knowledge the latter have never been analyzed within the context of the transition from exogenously to endogenously triggered saccades. As for the mean/median SRTs, the main present findings are that, as expected, Endo and EndoFC SRTs are longer than Exo SRTs by, in average, ∼27 ms, but that, contrary to previous reports [7], [18], mean Endo and EndoFC SRTs do not differ significantly. In fact, under blocked conditions, EndoFC SRTs were significantly shorter than Endo ones (by ∼10 ms), a shortening possibly due to the fact that under EndoFC blocked conditions observers could choose before the target onset the hemifield toward which they will direct the saccade. If one goes by Donders’ [60] classical (but strongly debated [61], [62]) serial/additive account of a choice RT (i.e. stimulus detection+stimulus discrimination+response selection+motor execution), one must conclude that the timings of the ‘response selection’ stage in Endo and EndoFC conditions are strictly equivalent.

The log-normal (but also linear-normal) fits of the presently assessed raw and vincentized SRT-distributions do not show any systematic uni- vs. bimodality partitioning. In particular, their analyses do not show any systematic tendency of bimodality for either the gap (compared with the non-gap) conditions or the Endo and EndoFC (compared with the Exo) conditions. This was attested by measures of (a) the AIC goodness of fit indices [54] for each observer and for each of the 36 experimental conditions and (b) the bimodality coefficient *β* [56] in conjunction with (c) the *excess kurtosis* test [57] (the latter two measured for the raw distributions only). In addition, the proportion of ‘fast’ (and, respectively, ‘slow’) SRTs under the bimodal fits did not show any systematic partitioning either between gap and no-gap conditions or between the three saccade triggering modes. In short, the only presently observed difference between reflexive and voluntary saccades was a global latency lengthening (of ∼27 ms) for the latter with no significant latency difference between Endo and EndoFC saccades. The most parsimonious account of this global result is that processes generating voluntary saccades, at least as operationalized here, do not differ from those generating reflexive ones other than in that only the former involve a binary choice sustained by competing, mutually inhibitory evidence accumulating processes [11], [12], [36], [37]. Alternatively, the reflexive and voluntary saccade generating processes may well be subtended by distinct brain structures (see the Introduction) but their preferential triggering task and stimuli may not have univocal effects so that reflexive and voluntary responses are randomly intermingled over trials. A third possibility would be that the dichotomy between the two response modes (and a fortiori between Endo and EndoFC actions) is conceptually unwarranted or at least poorly conceptualized [63]. Any of these putative accounts should be moderated by the general notion that the null hypothesis can never be formally accepted.

A more focal test of the putative difference between Endo and EndoFC saccades consisted in comparing the timings of the assumed inhibitory processes triggered by the distractor in the Endo task and by the alternative target in the EndoFC task. This comparison consisted in calculating for each time-bin the proportional change of the saccades number in the Endo and EndoFC SRT-distributions relative to their number in the corresponding baseline, Exo SRT-distribution [14], [29], [30]. The timings of the inhibition start and of its apex did not show any systematic difference between Endo and EndoFC conditions suggesting the existence of a unique inhibitory process whether triggered by a distractor or by an alternative target. This conclusion was supported by a correlation analysis between the inhibition points for each observer under Endo and EndoFC conditions (Table 3). In addition to the obligatory difference between the inhibition start and maximum time-points, the statistical analysis of these derived timings showed a significant effect of the ISI factor. Table 4 recapitulates these time-points together with the mean-SRTs for the Endo and EndoFC conditions.

**Table 4:** Mean SRTs and inhibition time-points for the Endo and EndoFC conditions.

	SRT			Inhibition time-points					
ISI	Endo	EndoFC	Mean	Endo		EndoFC		Mean	Mean
				Start	Max	Start	Max	Start	Max
−200	241	233	**237**	141	146	138	151	**139**	**148**
0	202	201	**201**	109	112	103	110	**106**	**111**
+200	176	168	**172**	79	89	75	90	**77**	**89**

The remarkable observation from Table 4 is that the inhibition from the distractor (Endo) or from the alternative target (EndoFC) starts (and reaches its maximum) earlier in time as the ISI increases from −200 to 0 to +200 ms by more or less the same amount as the SRTs shorten, namely by 33 and 29 ms for the inhibition start (or by 37 and 22 ms for the inhibition max) and by 36 and 29 ms for the SRTs shortening. This suggests that the mutual inhibition between target and distractor (or alternative target) is itself inhibited by the fixation neurons. More generally, the equivalence between the inhibition time-points under the present Endo and EndoFC conditions points once and again at the non-discriminability of these two saccade triggering modes.

### Discrepancies between Present and Past Studies

#### Exo-Endo-EndoFC SRT differences

In the present study the overall Exo-Endo SRT difference was ∼30 ms. Prior studies reported larger differences, e.g., ∼60 [4] and ∼100 ms [6]. In both studies (as in many others) the Exo condition was obtained by using a sharp luminance increment of one among two already present dimmer discs disposed symmetrically about fixation, while the Endo condition used the same dim disks with one of them pointed at by a central arrow. As SRTs (like manual reaction times) are known to depend on the amplitude of such transients [49], the larger Exo-Endo SRT difference in studies contrasting saccades to peripheral luminance transients with saccades directed by central symbolic cues comes as no surprise. Exo-Endo mean SRT differences reported in studies having used stimulation conditions similar to the present ones [11], [12] are indeed similar to those presently reported.

The global experimental design could also account for the presently assessed equivalence between Endo and EndoFC SRTs to be contrasted with the large difference (48 ms) between these two saccade triggering modes reported in [18]. In their study Endo saccades were guided by a central arrow pointing at one of two visible targets (just as in the studies above), while EndoFC saccades were obtained on trials where the arrow pointed away from both targets. Moreover, before the saccade was executed, a “change” or “no-change” central cue instructed subjects either to continue with their plan or to execute a saccade to the opposite target instead. Such uncertainty between saccade and anti-saccade response modes might have interfered more with the ‘free choice’ than with the stimulus guided process. In the absence of such uncertainty, Leach & Carpenter [17] have reported SRT differences between Exo and (pseudo-)EndoFC saccades (see Introduction) of only ∼30 ms, just like in the present study. That EndoFC saccades could be more prone than Endo saccades to interference by delayed counteracting instructions does not necessarily imply that Endo and EndoFC saccades are triggered by different mechanisms. It may well be a general case (to be further studied) that decision processes involving pre-specified vs. free chosen goals are more susceptible to interference by an imposed goal change.

A general denominator of most studies having contrasted reflexive and voluntary saccades (with the latter including anti-saccades) is their use of only two fixed target/distractor locations symmetrical about fixation. In the present study these locations were neither fixed nor symmetrical about fixation. It has been hypothesized and shown that increasing target location uncertainty prevents the advanced motor preparation (presumably initiated at the fixation offset in the gap-paradigm) and thereby makes express saccades vanish while attenuating the gap effect [27], [59]. The impediment to a motor preparatory activity in the present experimental conditions is most likely another factor accounting for the difference from other studies having contrasted reflexive and voluntary saccades.

Pesaran, Nelson & Andersen [64] reported higher correlations in spike and field potential activity between the dorsal premotor area (PMd) and the parietal reach region (PRR) in a reaching search task when monkeys were free to make their reaching choices than when they had to follow a predefined search sequence. They propose the existence of a specific fronto-parietal (free choice) decision circuit not activated in pre-instructed sensory-motor decisions. These authors’ stimulus design and experimental paradigm was not only very different from the present one but their two visual stimulation modes differed significantly raising doubts as to the critical factor subtending their differential results. Be it as it may, the literature is replete with examples showing tight task-dependent long-range correlations between a variety of areas presumably involved with decision processes [65].

In short, the discrepancies between some aspects of the present results and previous ones can be easily accounted for by differences in stimulation conditions (number of saccade target locations, target eccentricity, size, saliency, etc.) including those designed to define the three different saccade triggering modes.

## Conclusion

This is the first study to our knowledge having used a coherent transition from Exo to Endo to EndoFC saccade triggering modes and having compared them within a unified experimental paradigm using the same stimuli and observers. The assessed characteristics of endogenously triggered mandatory and ‘free choice’ saccades, as presently defined, do not differ significantly. This may encourage one to think that the very concept of ‘free choice’ might be vain. Data analysis also suggests that the mutual inhibition between a mandatory target and a distractor or between two possible targets is itself inhibited by the fixation units accounting for the gap-effect literature.

## Supporting Information

Figure S1 Individual Saccade Reaction Time (SRT) distributions and their best uni- or bimodal log-normal fits for the three saccade triggering modes (Exo, Endo and EndoFC) in the Blocked design. Inter-Stimulus Interval (ISI) = −200 ms, Target Duration (TD) = 100 ms.
(TIF)

Figure S2 As in Fig. S1 but for TD = 200 ms.
(TIF)

Figure S3 As in Fig. S1 but for ISI = 0 ms and TD = 100 ms.
(TIF)

Figure S4 As in Fig. S3 but for TD = 200 ms.
(TIF)

Figure S5 As in Fig. S3 but for TD = 100 ms.
(TIF)

Figure S6 As in Fig. S1 but for ISI = 200 ms.
(TIF)

Figure S7 Individual Saccade Reaction Time (SRT) distributions and their best uni- or bimodal log-normal fits for the three saccade triggering modes (Exo, Endo and EndoFC) in the Mixed design. Inter-Stimulus Interval (ISI) = −200 ms, Target Duration (TD) = 100 ms.
(TIF)

Figure S8 As in Fig. S7 but for TD = 200 ms.
(TIF)

Figure S9 As in Fig. S7 but for ISI = 0 ms and TD = 100 ms.
(TIF)

Figure S10 As in Fig. S9 but for TD = 200 ms.
(TIF)

Figure S11 As in Fig. S9 but for TD = 100 ms.
(TIF)

Figure S12 As in Fig. S7 but for ISI = 200 ms.
(TIF)

## References

[1] Kopecz K (1995) Saccadic reaction times in gap/overlap paradigms: a model based on integration of intentional and visual information on neural, dynamic fields. Vision Res 35(20): 2911–2925. 8533331 10.1016/0042-6989(95)00066-9

[2] Forbes K, Klein RM (1996) The magnitude of the fixation offset effect with endogenously and exogenously controlled saccades. J Cogn Neurosci 8(4): 344–352. 23971505 10.1162/jocn.1996.8.4.344

[3] Klein RM, Shore DI (2000) Relationships among modes of visual orienting In: Monsell S, Driver J, editors. *Attention and performance: XVIII Control of cognitive processes*,. Cambridge MA: MIT Press. 195–208.

[4] Mort DJ, Perry RJ, Mannan SK, Hodgson TL, Anderson E, et al (2003) Differential cortical activation during voluntary and reflexive saccades in man. Neuroimage 18(2): 231–246. 12595178 10.1016/s1053-8119(02)00028-9

[5] Munoz DP, Everling S (2004) Look away: the anti-saccade task and the voluntary control of eye movement. Nat Rev Neurosci 5(3): 218–228. 14976521 10.1038/nrn1345

[6] Walker R, McSorley E (2006) The parallel programming of voluntary and reflexive saccades. Vision Res 46(13: 2082–93. 16473385 10.1016/j.visres.2005.12.009

[7] Kennard C, Mannan SK, Nachev P, Parton A, Mort DJ, et al (2005) Cognitive processes in saccade generation. Ann N Y Acad Sci 1039(1): 176–83. 15826972 10.1196/annals.1325.017

[8] Walker R, Walker DG, Husain M, Kennard C (2000) Control of voluntary and reflexive saccades. Exp Brain Res 130(4): 540–544. 10717796 10.1007/s002219900285

[9] Matsumora T, Koida K, Komatsu H (2008) Relationship Between Color Discrimination and Neural Responses in the Inferior Temporal Cortex of the Monkey. J Neurophysiol 100: 3361–3374. 18922950 10.1152/jn.90551.2008

[10] Stanford TR, Shankar S, Massoglia DP, Costello GM, Salinas E (2010) Perceptual decision making in less than 30 milliseconds. Nat Neurosci 13(3): 379–385. 20098418 10.1038/nn.2485 PMC2834559

[11] Walker R, Deubel H, Schneider WX, Findlay JM (1997) Effect of Remote Distractors on Saccade Programming: Evidence for an Extended Fixation Zone. J Neurophysiol 78(2): 1108–1119. 9307138 10.1152/jn.1997.78.2.1108

[12] Walker R, Kentridge RW, Findlay JM (1995) Independent contributions of the orienting of attention, fixation offset and bilateral stimulation on human saccadic latencies. Exp Brain Res 103: 294–310. 7789437 10.1007/BF00231716

[13] Watanabe K, Funahashi S (2007) Prefrontal delay-period activity reflects the decision process of a saccade direction during a free-choice ODR task. Cereb Cortex 17: 88–100. 10.1093/cercor/bhm102 17726006

[14] Reingold EM, Stampe DM (2002) Saccadic inhibition in voluntary and reflexive saccades. J Cogn Neurosci 14(3): 371–388. 11970798 10.1162/089892902317361903

[15] Cotti J, Panouilleres M, Munoz DP, Vercher J-L, Pélisson D, et al (2009) Adaptation of reactive and voluntary saccades: different patterns of adaptation revealed in the antisaccade task. J Physiol 587(1): 127–138. 19015199 10.1113/jphysiol.2008.159459 PMC2670028

[16] Wurtz RH, Goldberg ME (1972) Activity of superior colliculus in behaving monkey 3 Cells discharging before eye movements. J Neurophysiol 35: 575–586. 4624741 10.1152/jn.1972.35.4.575

[17] Leach JCD, Carpenter RHS (2001) Saccadic choice with asynchronous targets: evidence for independent randomisation. Vision Res 41: 3437–3445. 11718785 10.1016/s0042-6989(01)00059-1

[18] Nachev P, Rees G, Parton A, Kennard C, Husain M (2005) Volition and Conflict in Human Medial Frontal Cortex. Curr Biol 15(1): 122–128. 15668167 10.1016/j.cub.2005.01.006 PMC2648721

[19] Henik A, Rafal R, Rhodes D (1994) Endogenously generated and visually guided saccades after lesions of the human frontal eye fields. J Cogn Neurosci 6(4): 400–411. 23961734 10.1162/jocn.1994.6.4.400

[20] Pierrot-Deseilligny C, Rivaud S, Gaymard B, Agid Y (1991) Cortical control of reflexive visually-guided saccades. Brain 114 (3): 1473–85. 10.1093/brain/114.3.1473 2065261

[21] Pierrot-Deseilligny C, Rivaud S, Gaymard B, Agid Y (1991) Cortical control of memory-guided saccades in man. Exp Brain Res 83: 607–617. 2026201 10.1007/BF00229839

[22] Schiller PH, Chou I (2000) The effects of anterior arcuate and dorsomedial frontal cortex lesions on visually guided eye movements: 2. Paired and multiple targets. Vision Res 40(10–12): 1627–1638. 10788662 10.1016/s0042-6989(00)00058-4

[23] Brown MR, DeSouza JF, Goltz HC, Ford K, Menon RS, et al (2004) Comparison of memory- and visually guided saccades using event-related fMRI. J Neurophysiol 91(2): 873–889. 14523078 10.1152/jn.00382.2003

[24] Nachev P, Kennard C, Husain M (2008) Functional role of the supplementary and pre-supplementary motor areas. Nat Rev Neurosci 9(11): 856–69. 18843271 10.1038/nrn2478

[25] Schall JD (2001) Neural basis of deciding, choosing and acting. Nat Rev Neurosci 2(1): 33–42. 11253357 10.1038/35049054

[26] Gaymard B, Ploner CJ, Rivaud-Péchoux S, Pierrot-Deseilligny C (1999) The frontal eye field is involved in spatial short-term memory but not in reflexive saccade inhibition. Exp Brain Res 129: 288–301. 10591903 10.1007/s002210050899

[27] Rolfs M, Vitu F (2007) On the limited role of target onset in the gap task: Support for the motor-preparation hypothesis. J Vision 7: 1–20. 10.1167/7.10.7 17997676

[28] Saslow MG (1967) Effects of components of displacement-step stimuli upon latency of saccadic eye movements. J Opt Soc Am 57: 1024–1029. 6035296 10.1364/josa.57.001024

[29] Bompas A, Sumner P (2011) Saccadic Inhibition Reveals the Timing of Automatic and Voluntary Signals in the Human Brain. J Neurosci 31(35): 12501–12512. 21880912 10.1523/JNEUROSCI.2234-11.2011 PMC6703251

[30] Reingold EM, Stampe DM (2004) Saccadic inhibition in reading. J Exp Psychol Hum Percept Perform 30(1): 194–211. 14769077 10.1037/0096-1523.30.1.194

[31] Fischer B, Weber H, Biscaldi M, Aiple F, Otto P, Stuhr V (1993) Separate populations of visually guided saccades in humans: reaction times and amplitudes. Exp Brain Res 4: 528–541. 10.1007/BF00229043 8454016

[32] Kirchner H, Thorpe SJ (2006) Ultra-rapid object detection with saccadic eye movements: visual processing speed revisited. Vision Res 46(11): 1762–1776. 16289663 10.1016/j.visres.2005.10.002

[33] Fischer B, Rampsperger E (1984) Human express saccades: extremely short reaction times of goal directed eye movements. Exp Brain Res 57: 191–195. 6519226 10.1007/BF00231145

[34] Cardoso-Leite P, Gorea A, Mamassian P (2007) Temporal order judgment and simple reaction times: Evidence for a common processing system. J Vision 7: 1–14. 10.1167/7.6.11 17685794

[35] Otto TU, Mamassian P (2012) Noise and correlations in parallel perceptual decision making. Curr Biol 22(15): 1391–1396. 22771043 10.1016/j.cub.2012.05.031

[36] Boucher L, Palmeri TJ, Logan GD, Schall JD (2007) Inhibitory control in mind and brain: an interactive race model of countermanding saccades. Psychol Rev 114(2): 376–97. 17500631 10.1037/0033-295X.114.2.376

[37] Purcell BA, Heitz RP, Cohen JY, Schall JD, Logan GD, et al (2010) Neurally constrained modeling of perceptual decision making. Psychol Rev 117(4): 1113–1143. 20822291 10.1037/a0020311 PMC2979343

[38] Trappenberg TP, Dorris MC, Munoz DP, Klein RM (2001) A model of saccade initiation based on the competitive integration of exogenous and endogenous signals in the superior colliculus. J Cogn Neurosci 13(2): 256–271. 11244550 10.1162/089892901564306

[39] Marino RA, Trappenberg TP, Dorris MC, Munoz DP (2012) Spatial interactions in the superior colliculus predict saccade behavior in a neural field model. J Cogn Neurosci 24(2): 315–336. 21942761 10.1162/jocn_a_00139

[40] D L Sparks and R Hartwich-Young (1989) The deep layers of the superior colliculus. In Wurtz RH, Goldberg ME, editors. *The neurobiology of saccadic eye-movements*. Amsterdam: Elsevier. 213–255.

[41] Gezeck S, Fischer B, Timmer J (1997) Saccadic reaction times: a statistical analysis of multimodal distributions. Vision Res 37(15): 2119–2131. 9327059 10.1016/s0042-6989(97)00022-9

[42] Gezeck S, Timmer J (1998) Detecting multimodality in saccadic reaction time distributions in gap and overlap tasks. Biol Cybern 78(4): 293–305. 9652079 10.1007/s004220050434

[43] Dorris MC, Munoz DP (1995) A neural correlate for the gap effect on saccadic reaction times in monkey. J Neurophysiol 73(6): 2558–2562. 7666161 10.1152/jn.1995.73.6.2558

[44] Dorris MC, Paré M, Munoz DP (1997) Neuronal activity in monkey superior colliculus related to the initiation of saccadic eye movements. J Neurosci 17(21): 8566–8579. 9334428 10.1523/JNEUROSCI.17-21-08566.1997 PMC6573744

[45] Munoz DP, Istvan PJ (1998) Lateral inhibitory interactions in the intermediate layers of the monkey superior colliculus. J Neurophysiol 79(3): 1193–209. 9497401 10.1152/jn.1998.79.3.1193

[46] Brainard DH (1997) The Psychophysics Toolbox. Spatial Vis 10: 433–436. 9176952

[47] Pelli DG (1997) The VideoToolbox software for visual psychophysics: Transforming numbers into movies. Spatial Vis 10: 437–442. 9176953

[48] Cornelissen FW, Peters EM, Palmer J (2002) The Eyelink Toolbox: eye tracking with MATLAB and the Psychophysics Toolbox. Behav Res Methods, Instruments, Comput 34(4): 613–617. 10.3758/bf03195489 12564564

[49] Boch R, Fischer B, Rampsperger E (1984) Express-Saccades of the monkey: reaction times versus intensity, size, duration, eccentricity of their targets. Exp Brain Res 55: 223–231. 6745363 10.1007/BF00237273

[50] Fischer B (1987) The Preparation of Visually Guided Saccades. Rev Physiol Biochem Pharmacol 106: 1–35. 3112911 10.1007/BFb0027574

[51] Fischer B, Gezeck S, Huber W (1995) The three-loop model: a neural network for the generation of saccadic reaction times. Biol Cybern 72(3): 185–196. 7703295 10.1007/BF00201483

[52] Ratcliff R (1979) Group Reaction Time Distributions and an Analysis of Distribution Statistics Psychol Bull. 86(3): 446–461. 451109

[53] Vincent SB (1912) The functions of the vibrissae in the behavior of the white rat. Behav Monogr 1(5).

[54] Akaike H (1974) A new look at the statistical model identification. IEEE Trans Automat Contr 19(6): 716–723.

[55] Hellwig B, Hengstler JG, Schmidt M, Gehrmann MC, Schormann W, et al (2010) Comparison of scores for bimodality of gene expression distributions and genome-wide evaluation of the prognostic relevance of high scoring genes. Bioinformatics 11(276): 1–18. 10.1186/1471-2105-11-276 PMC2892466 20500820

[56] Ellision AM (1987) Effect of seed dimorphism on the density-dependent dynamics of experimental populations of Atriplex triangularis (Chenopodiaceae). Am J Bot 74(8): 1280–1288.

[57] Muratov AL, Gnedin OY (2010) Modeling the Metallicity Distribution of Globular Clusters. Astrophys J 718(2): 1266–1288.

[58] Sheskin DJ (2003) Handbook of Parametric and Nonparametric Statistical Procedures, 3rd ed. New York: CRC Press.

[59] Paré M, Munoz DP (1996) Saccadic reaction time in the monkey: advanced preparation of oculomotor programs is primarily responsible for express saccade occurrence. J Neurophysiol 76(6): 3666–3681. 8985865 10.1152/jn.1996.76.6.3666

[60] Donders FC (1868/1969) On the speed of mental processes. Acta Psychol 30: 412–431. 10.1016/0001-6918(69)90065-1 5811531

[61] Miller J, Low K (2001) Motor Processes in Simple, Go/No-Go, Choice Reaction Time Tasks: A Psychophysiological Analysis. J Exp Psychol Hum Percept Perform 27(2): 266–289. 11318047

[62] Sternberg S (2001) Separate modifiability, mental modules, the use of pure and composite measures to reveal them. Acta Psychol 106(1–2): 147–246. 10.1016/s0001-6918(00)00045-7 11256336

[63] Nachev P, Husain M, Kennard C (2008) Volition and eye movements. In: Kennard C, Leigh RJ, editors. Progress in Brain Research Vol 171. New York: Elsevier. 391–398. 10.1016/S0079-6123(08)00657-2 18718331

[64] Pesaran B, Nelson MJ, Andersen RA (2008) Free choice activates a decision circuit between frontal and parietal cortex. Nature 453(7193): 406–409. 18418380 10.1038/nature06849 PMC2728060

[65] Pesaran B (2010) Neural correlations, decisions, actions Curr Opin Neurobiol. 20(2): 166–171. 10.1016/j.conb.2010.03.003 PMC2862782 20359885

