# Supplementary figures and images for: A Unified Comparison of Stimulus-Driven, Endogenous Mandatory and ‘Free Choice’ Saccades
Source: PLoS One. 2014 Feb 20;9(2):e88990. doi: 10.1371/journal.pone.0088990 (PMC3930601; doi:10.1371/journal.pone.0088990)

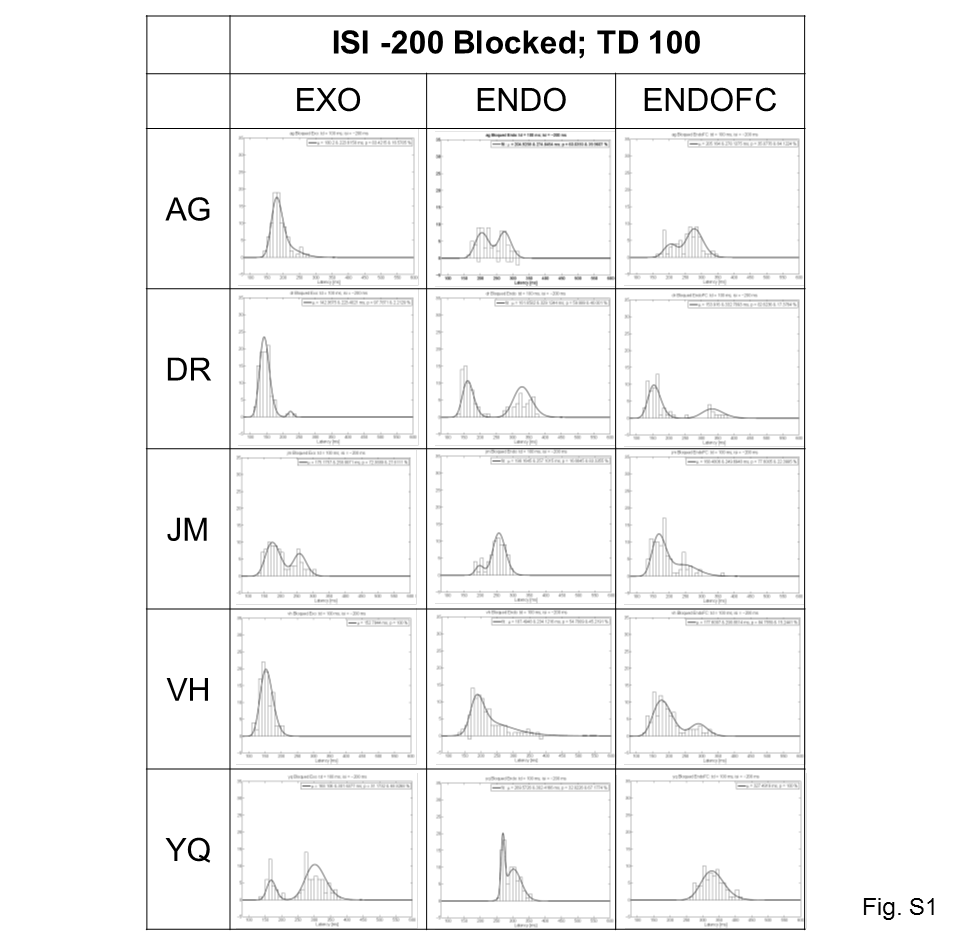

Supplement: Figure S1 — Individual Saccade Reaction Time (SRT) distributions and their best uni- or bimodal log-normal fits for the three saccade triggering modes (Exo, Endo and EndoFC) in the Blocked design. Inter-Stimulus Interval (ISI) = −200 ms, Target Duration (TD) = 100 ms. (TIF) [file pone.0088990.s001.tif]

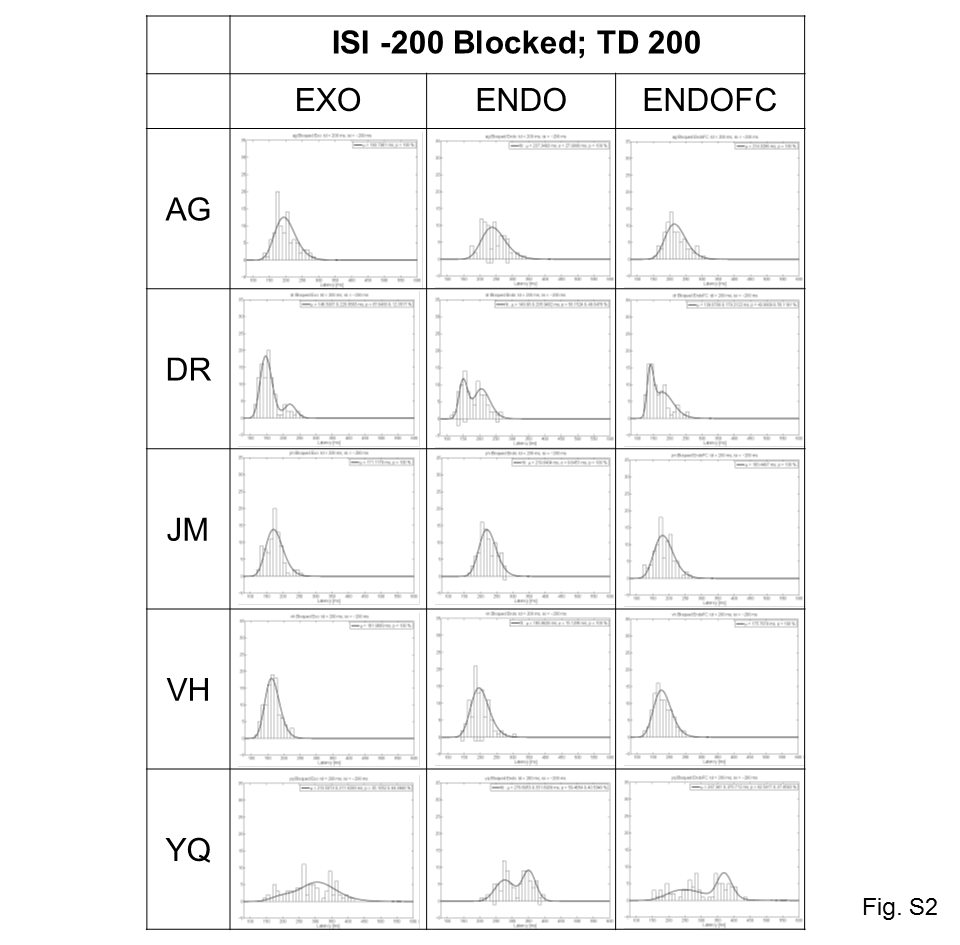

Supplement: Figure S2 — As in Fig. S1 but for TD = 200 ms. (TIF) [file pone.0088990.s002.tif]

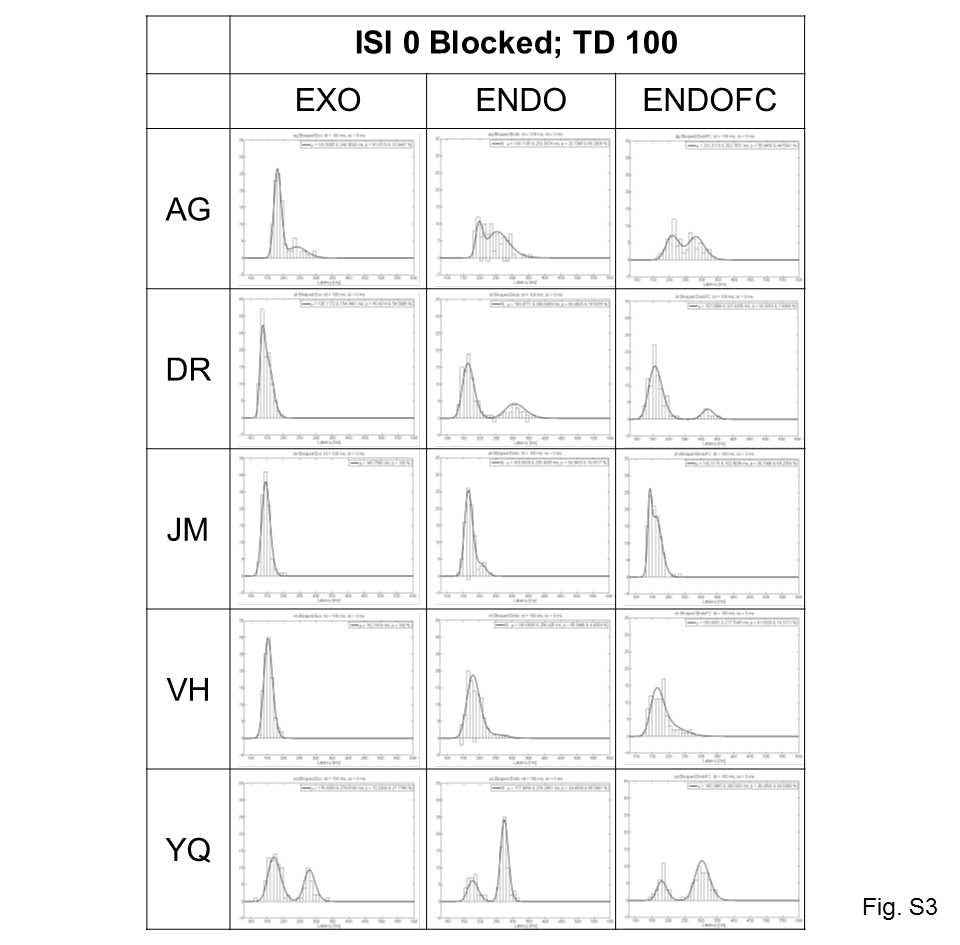

Supplement: Figure S3 — As in Fig. S1 but for ISI = 0 ms and TD = 100 ms. (TIF) [file pone.0088990.s003.tif]

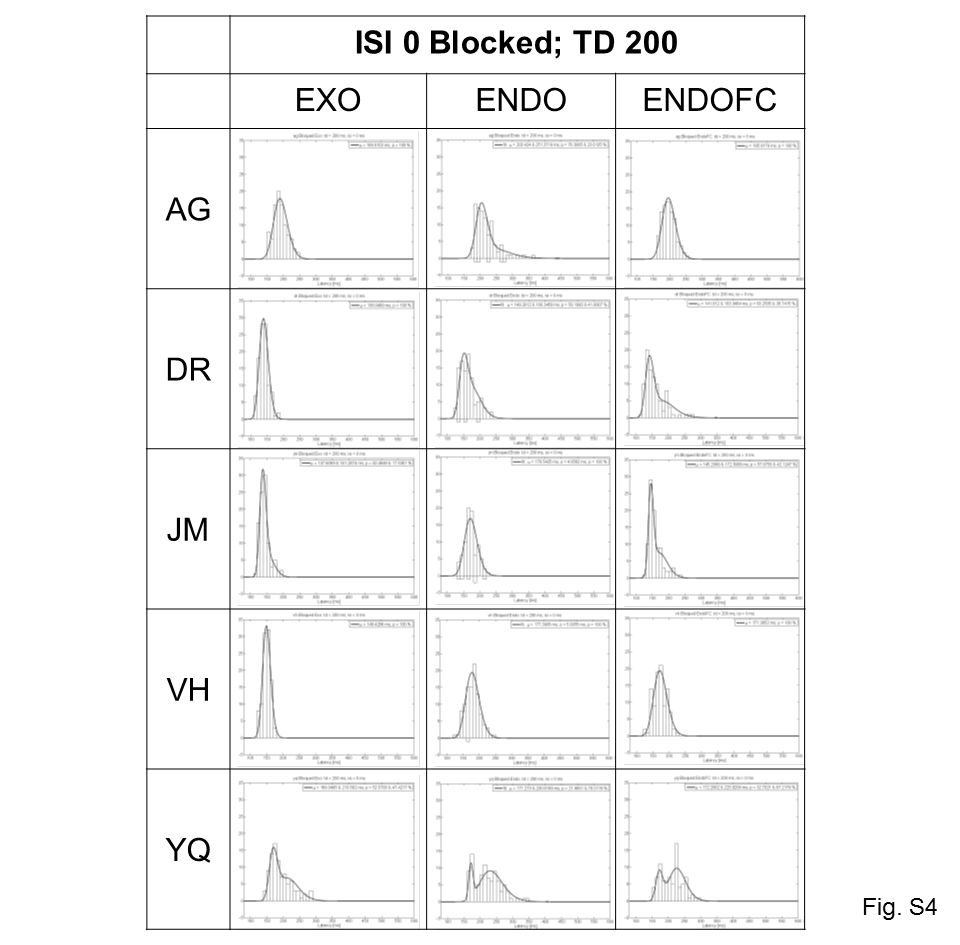

Supplement: Figure S4 — As in Fig. S3 but for TD = 200 ms. (TIF) [file pone.0088990.s004.tif]

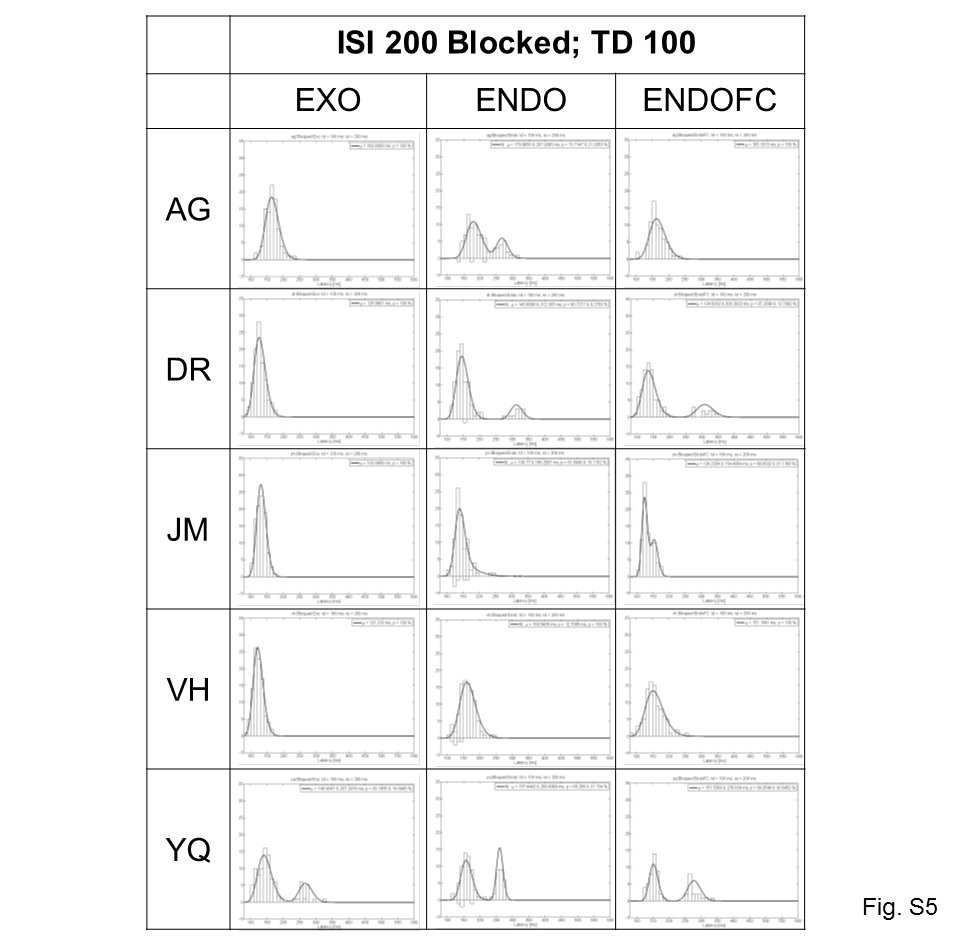

Supplement: Figure S5 — As in Fig. S3 but for TD = 100 ms. (TIF) [file pone.0088990.s005.tif]

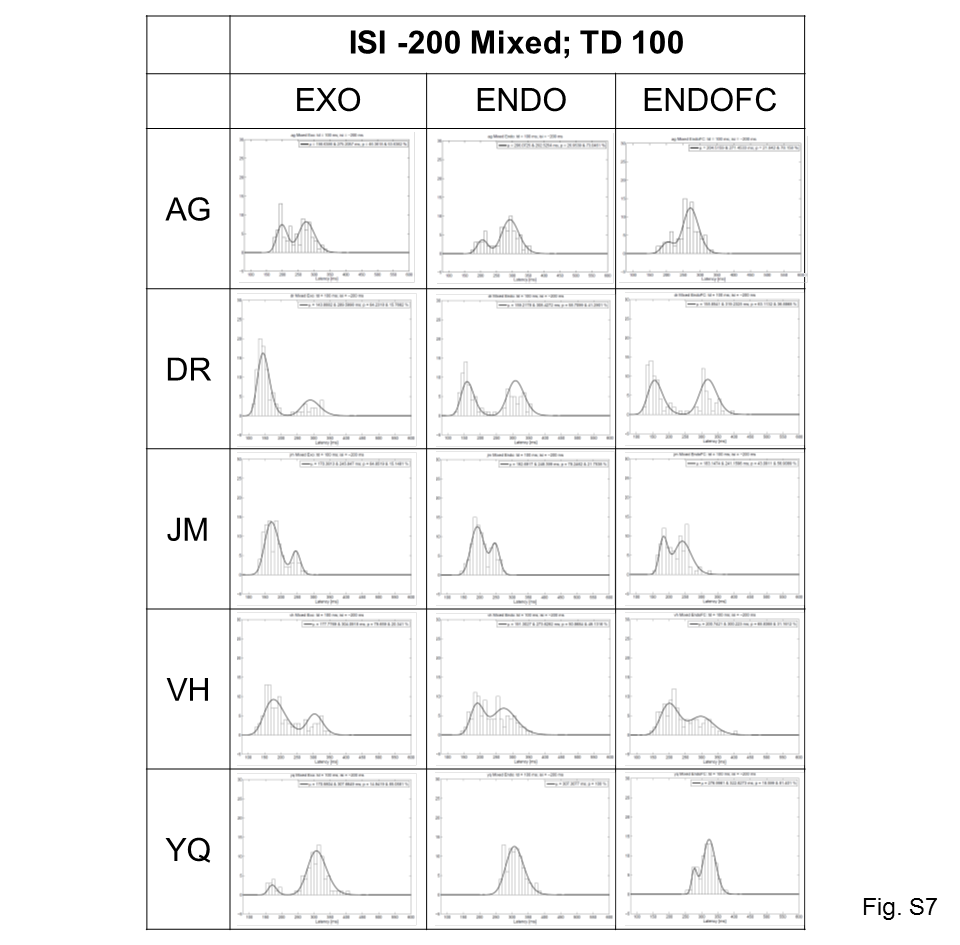

Supplement: Figure S6 — As in Fig. S1 but for ISI = 200 ms. (TIF) [file pone.0088990.s006.tif]

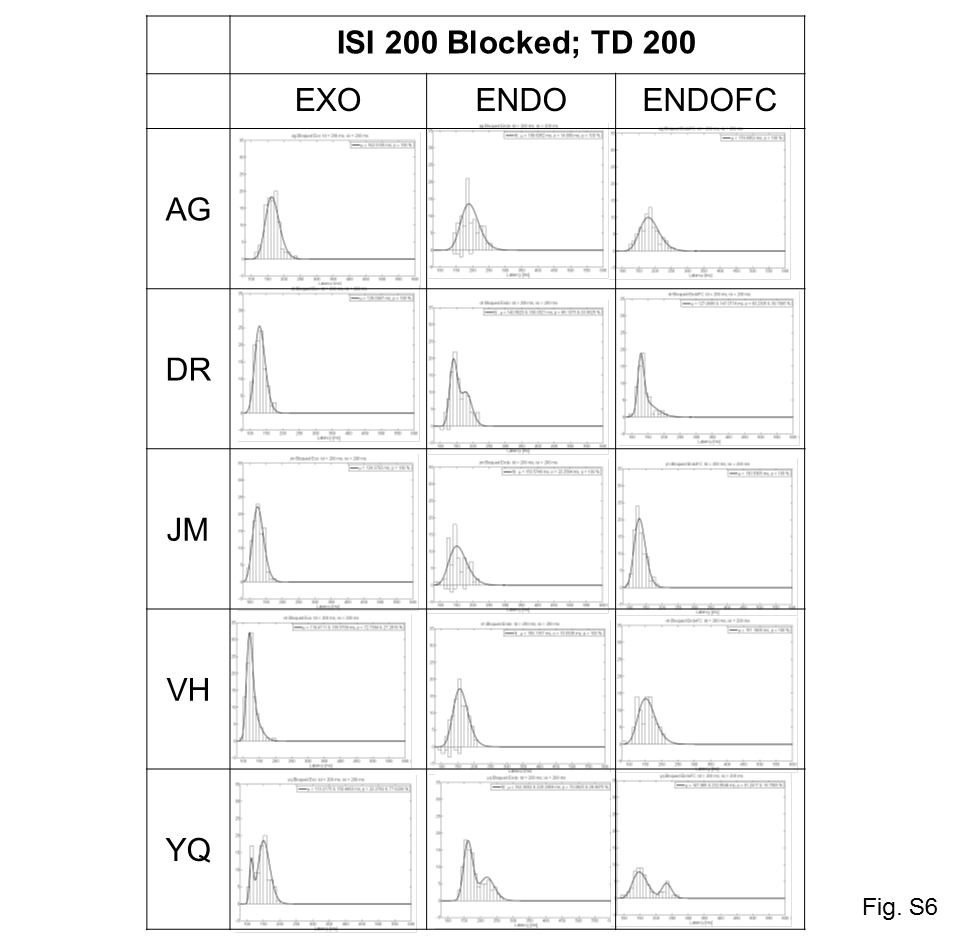

Supplement: Figure S7 — Individual Saccade Reaction Time (SRT) distributions and their best uni- or bimodal log-normal fits for the three saccade triggering modes (Exo, Endo and EndoFC) in the Mixed design. Inter-Stimulus Interval (ISI) = −200 ms, Target Duration (TD) = 100 ms. (TIF) [file pone.0088990.s007.tif]

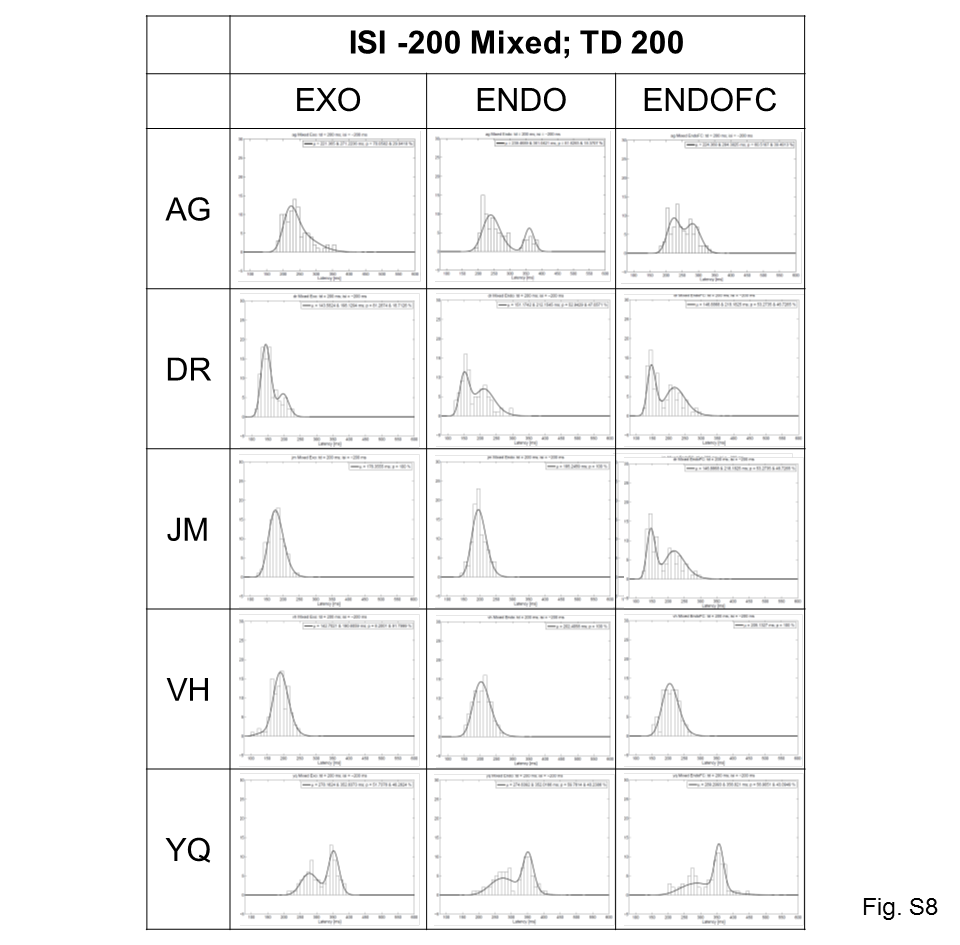

Supplement: Figure S8 — As in Fig. S7 but for TD = 200 ms. (TIF) [file pone.0088990.s008.tif]

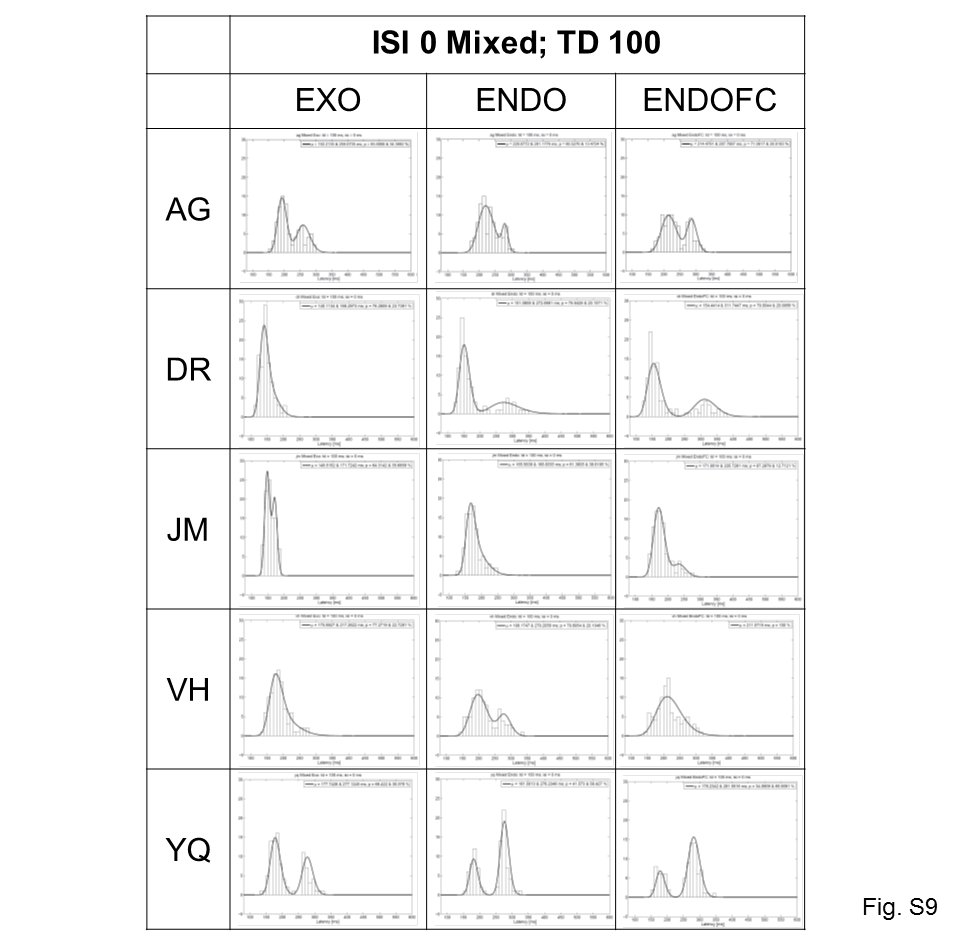

Supplement: Figure S9 — As in Fig. S7 but for ISI = 0 ms and TD = 100 ms. (TIF) [file pone.0088990.s009.tif]

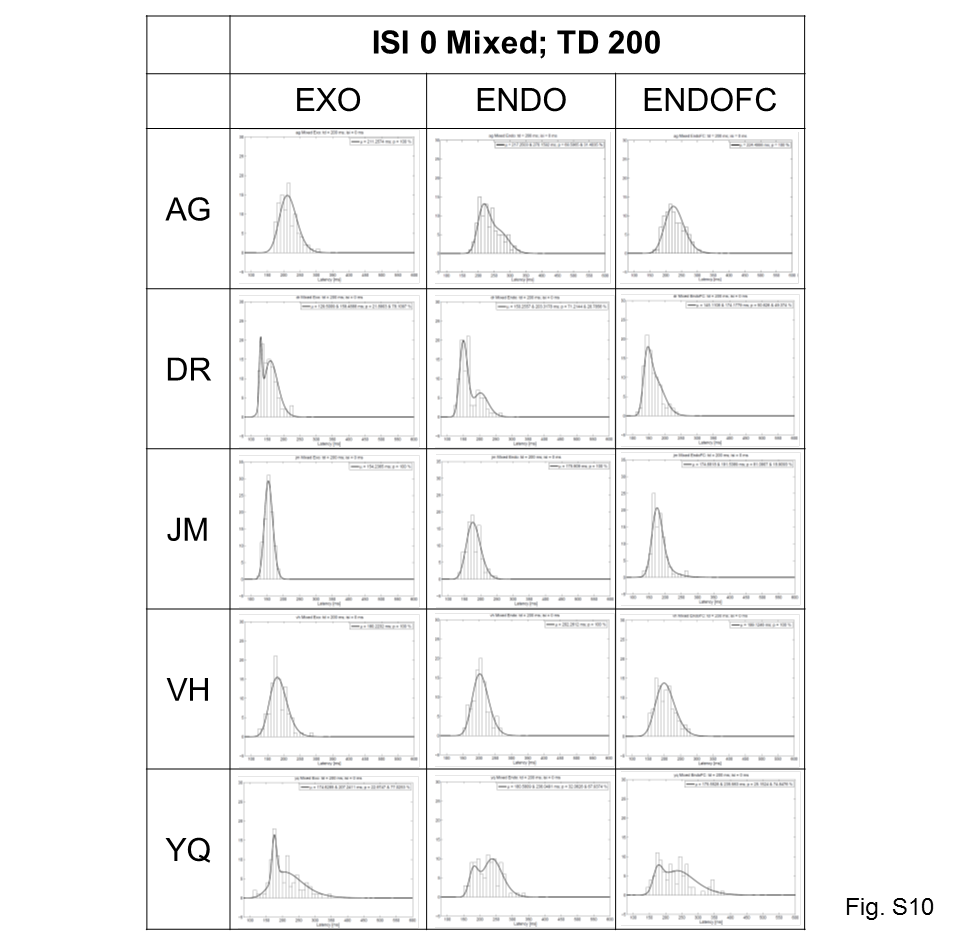

Supplement: Figure S10 — As in Fig. S9 but for TD = 200 ms. (TIF) [file pone.0088990.s010.tif]

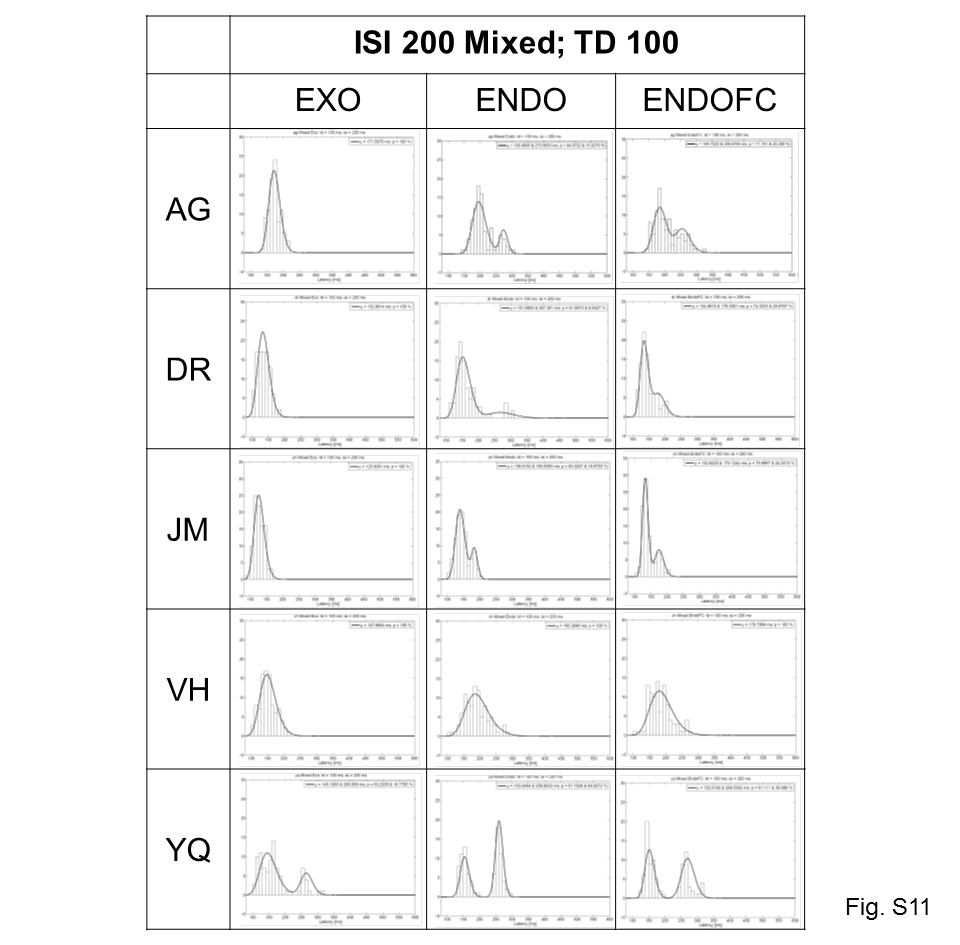

Supplement: Figure S11 — As in Fig. S9 but for TD = 100 ms. (TIF) [file pone.0088990.s011.tif]

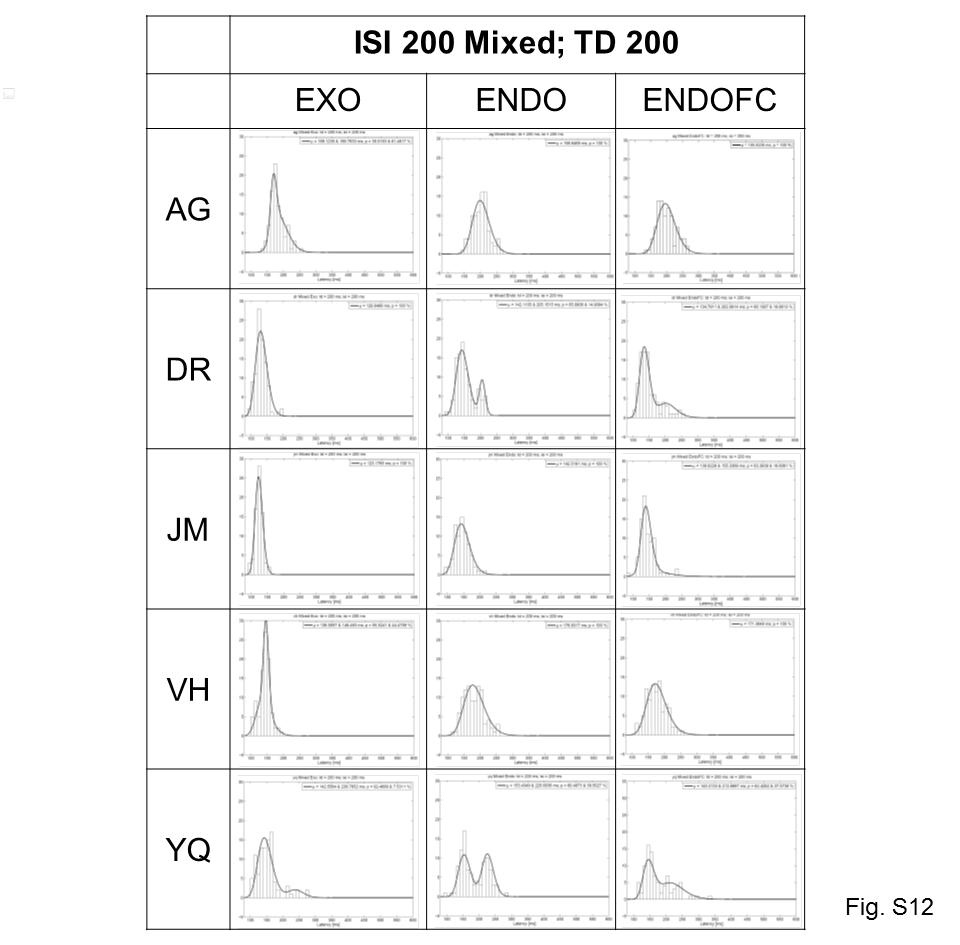

Supplement: Figure S12 — As in Fig. S7 but for ISI = 200 ms. (TIF) [file pone.0088990.s012.tif]
